# DNMT1 mediates the disturbed flow-induced endothelial to mesenchymal transition through disrupting β-alanine and carnosine homeostasis

**DOI:** 10.7150/thno.84427

**Published:** 2023-08-06

**Authors:** Jianan Zhao, Chuanrong Zhao, Fangfang Yang, Zhitong Jiang, Juanjuan Zhu, Weijuan Yao, Wei Pang, Jing Zhou

**Affiliations:** 1 Department of Physiology and Pathophysiology, School of Basic Medical Sciences; Hemorheology Center, School of Basic Medical Sciences, Peking University, Beijing 100191, China.; 2 State Key Laboratory of Vascular Homeostasis and Remodeling, Peking University, Beijing 100191, China.; 3 National Health Commission Key Laboratory of Cardiovascular Molecular Biology and Regulatory Peptides; Beijing Key Laboratory of Cardiovascular Receptors Research, Peking University, Beijing 100191, China.

**Keywords:** Endothelial dysfunction, DNA methylation, β-alanine, Carnosine, Hemodynamic disturbed flow

## Abstract

**Background:** Increasing evidence suggests that hemodynamic disturbed flow induces endothelial dysfunction via a complex biological process so-called endothelial to mesenchymal transition (EndoMT). Recently, DNA methyltransferases (DNMTs) was reported as a key molecular mediator to promote EndoMT. Our understanding of how DNMTs, particularly the maintenance DNMTs, DNMT1, coordinate EndoMT is still lacking.

**Methods:** A parallel-plate flow apparatus and perfusion devices were used to apply fluid with endothelial protective pulsatile shear (PS, to mimic the laminar flow) or harmful oscillatory shear (OS, to mimic the disturbed flow) to cultured endothelial cells (ECs). Endothelial lineage tracing mice and conditional EC Dnmt1 knockout mice were subjected to a surgery of carotid partial ligation to generate the flow-accelerated atherogenesis models. Western blotting, quantitative RT-PCR, immunofluorescent staining, methylation-specific PCR, chromatin immunoprecipitation, endothelial functional assays, and assessments for neointimal formation and atherosclerosis were performed.

**Results:** Inhibition of DNMTs with 5-aza-2'-deoxycytidine (5-Aza) suppressed the disturbed flow/OS-induced EndoMT, both in cultured cells and the endothelial lineage tracing mice. 5-Aza also ameliorated the downregulation of aldehyde dehydrogenases (ALDHs) and β-alanine biosynthesis caused by disturbed flow/OS. Knockdown of the ALDH family proteins, ALDH2, ALDH3A1, and ALDH6A1, showed an EndoMT-induction effect as OS. Supplementation of cells with the functional metabolites of β-alanine, carnosine and acetyl-CoA (acetate), reversed EndoMT, likely via inhibiting the phosphorylation of Smad2/3. Endothelial-specific knockout of Dnmt1 protected the vasculature from disturbed flow-induced remodeling and atherosclerosis.

**Conclusions:** Endothelial DNMT1 acts as one of the key epigenetic factors to mediate the hemodynamically regulated EndoMT at least through repressing the expression of ALDH2, ALDH3A1, and ALDH6A1. Supplementation with carnosine and acetate may have a great potential in the prevention and treatment of atherosclerosis.

## Introduction

Endothelial-to-mesenchymal transition (EndoMT) is a cellular reprogramming process in which endothelial cells (ECs) undergoes a series of molecular events that lead to changes in phenotype toward mesenchymal cells such as myofibroblasts and smooth muscle cells (SMCs) 1. Emerging studies suggest that EndoMT contributes to the development and progression of various cardiovascular diseases, including atherosclerosis, pulmonary hypertension, valvular disease, and fibroelastosis 2. EndoMT may be induced by the environmental chemical (e.g., transforming growth factor-beta 1, TGF-β1) or mechanical stimuli (e.g., the disturbed fluid shear stress) 3. ECs lining the inner wall of the vasculature are highly sensitive to hemodynamic shear stress that acts on the luminal surface in a tangential manner. In the straight segments of the arterial trees, ECs experience a stable high-shear laminar flow that is essential for maintaining their physiological functions 4. In contrast, in the arterial branches and curvatures, ECs are exposed to an unstable disturbed blood flow that produces a low and oscillatory shear stress, resulting in endothelial dysfunction and atherogenesis 5-8. Accumulating evidence demonstrates a pivotal role of disturbed shear stress in activating EndoMT 9-12. However, the underlying mechanisms remain to be adequately elucidated.

Lai B et al. reported epigenetic regulation via DNA methylation to be a key mechanism involved in the shear stress-regulated EndoMT 13. DNA methylation is the most stable epigenetic modification involving the covalent transfer of a methyl group to the C-5 position of a cytosine base pair that occurs most often in a cytosine-guanine dinucleotide 14, 15. DNA methyltransferases (DNMTs) catalyze the transfer of a methyl group from donor S-adenyl-l-methionine to cytosine, generating 5-methylcytosine (5-mC) 16. DNMT1 is canonically referred to as a maintenance methyltransferase that mains DNA methylation after DNA replication, although it also has de novo methylation capabilities 15, 16. DNMT3A and DNMT3B are responsible for de novo establishment of DNA methylation patterns 15, 17. Studies from us and others have shown that the expression of DNMT1 and DNMT3A, but not DNMT3B, were upregulated by disturbed flow in ECs *in vivo* and *in vitro* 15, 18-21. The shear-induced upregulation of DNMT1 is revealed in our prior study to be mediated by integrin β_3_-Shc/FAK-ERK1/2-mTOR mechanotransduction signaling 19. In experimental shear flow models, compared with the pulsatile shear (PS) that simulates the laminar flow, oscillatory shear (OS) simulating the disturbed flow increased the DNA methylation of the promoter regions of EC marker (e.g., von Willebrand factor (VWF), CD31, and cadherin 5 (CDH5) genes) but decreased that of mesenchymal cell markers (e.g., cadherin 2 (CDH2), fibroblast-specific protein 1 (FSP1), and vimentin), hinting at a direct regulation of DNA methylation by DNMTs on the EndoMT marker genes 13. As DNMTs were reported to be responsible for methylation of regulatory genes upstream of those EndoMT markers 22-24, we speculated that disturbed shear stress may also activate EndoMT through DNMTs-mediated hypermethylation of novel central players rather than the functional EndoMT markers, despite of the fact that such mechanisms are rarely documented.

Aberrant endothelial metabolism seemed to play a role in EndoMT 2, 25. Supplementation of colorectal cancer cells with carnosine inhibited their epithelial-to-mesenchymal transition (EMT) 26, a process similar to EndoMT. The underlying mechanistic insight was suggested by reports showing that carnosine decreased the phosphorylation level of Smad2 25, 27 that mediates the TGF-β signaling pathway, a central player in EndoMT 28. Carnosine (beta-alanyl-L-histidine) is a histidine-containing dipeptide made up of the amino acids β-alanine and L-histidine. It is abundant in skeletal and cardiac muscles, liver tissues, and regions of the central nervous system 29, and is known to be important for maintaining redox homeostasis due to its antioxidant activity 30. For these reasons, carnosine is recognized as a protective factor in muscle cells, nerve cells, and ECs 31-33. Since L-histidine is more abundant in various tissues than β-alanine, the latter is considered to be the rate-limiting precursor of endogenous synthesis of carnosine 34. The aldehyde dehydrogenase (ALDH) superfamily consists of a wide variety of enzymes responsible of oxidizing numerous aldehydes to their corresponding nontoxic carboxylic acids 35, being involved in detoxification, biosynthesis, antioxidant, structural and regulatory functions 36. Dysregulation of ALDHs has been linked to vascular diseases and, in particular, atherosclerosis. Among ALDHs, ALDH2, ALDH3A1, and ALDH6A1 are known for their critical role in β-alanine metabolism. Specifically, ALDH2 and ALDH3A1 are required for biosynthesis of β-alanine 37, while ALDH6A1 catalyzes the generation of an important metabolic intermediate, acetyl-CoA, from malonate semialdehyde that can ben biosynthesized from β-alanine 37. Intriguingly, lower acetyl-CoA levels also cause EndoMT through increasing the phosphorylation level of Smad2 25. Whether β-alanine metabolism and the ALDH family play a role in shear stress-regulated EndoMT has not been reported so far.

In the present study, we demonstrated an important role of β-alanine and carnosine metabolism in regulating the endothelial function and phenotype. The disturbed flow-upregulated endothelial DNMT1 inhibits the expression of ALDH2, ALDH3A1, and ALDH6A1 through promoting their promoter methylation; dysregulation of ALDH2, ALDH3A1, and ALDH6A1 results in impairment in β-alanine, carnosine, and acetyl-CoA biosynthesis, leading to increased Smad2/3 phosphorylation and activation of the TGF-β signaling that ultimately causes EndoMT and atherosclerosis. Our results indicate that endothelial β-alanine metabolism is required for maintaining EC hemostasis and that therapeutic targeting endothelial metabolism could provide the basis for treating EndoMT-linked vascular diseases.

## Methods

### Cell culture

Human umbilical vein endothelial cells (HUVECs) were isolated from umbilical cords from healthy patients after full-term deliveries. Umbilical cords were obtained with the agreement of the patients and approved by the Peking University People's Hospital Medical Ethics Committee (2015PHB024). HUVECs within passages 5-8 were maintained in Medium 199 supplemented with 10% fetal bovine serum (FBS) (Hyclone, SV30087), endothelial cell growth factor (ECGF) (4 g/mL) (Sigma, E1388), 1% penicillin/streptomycin (Yeasen, 60162ES76) at 37 ℃ in an incubator with 95% humidified air and 5% CO_2_ and passaged every 3 days.

Immortalized human vascular endothelial cells (EA. hy926) were obtained from ZOMANBIO (ZKC1051-1) and maintained in Dulbecco's Modified Eagle Medium (DMEM, MACGENE, CM10013) supplemented with 10% fetal bovine serum (FBS) (Gemini, 900-008), 1% penicillin/streptomycin at 37 ℃ in an incubator with 95% humidified air and 5% CO_2_ and passaged every 3 days.

### Flow experiments

Monocultured HUVECs seeded on collagen I (50 µg/mL)-coated glass slides were subjected to shear stress in a parallel-plate flow apparatus as described previously 38. The flow channel in the chamber was created by a silicon gasket with dimensions of 2.5 cm in width (w), 5.0 cm in length, and 0.025 cm in height (h). The chamber containing the cell-seeded glass slide fastened with the gasket was connected to a perfusion loop system, kept in a constant temperature-controlled enclosure, with pH maintained at 7.4 by continuous gassing with a humidified mixture of 5% (vol/vol) CO_2_, 20% (vol/vol) O_2_, and 75% (vol/vol) N_2_. The shear stress generated on the HUVECs was estimated as 6Qμ/wh^2^, where Q is flow rate, w is dimension in width and μ is perfusate viscosity. The flow of pulsatile shear (PS, 12 ± 4 dynes/cm^2^) or oscillatory shear (OS, 0.5 ± 4 dynes/cm^2^) is composed of mean flow with shear stress at 12 dynes/cm^2^ or 0.5 dynes/cm^2^ supplied by a sinusoidal oscillation using a piston pump with a frequency of 1 Hz and a peak-to-peak amplitude of ± 4 dynes/cm^2^. HUVECs were perfused in M199 medium containing 2% FBS for the indicated time.

### Endothelial migration assay

Use the tip of 1mL pipette to make a scratch on the HUVEC monolayers at the bottom of the seed slide or dish, and then replace the complete medium with a serum-reduced medium (2%). Wound closure was monitored by microscope at 0 and 12 hours after allowing the cells to migrate. Image J software was used to measure and calculate the wound closure rate. Representative images were selected to most accurately represent the group mean/average of all available data.

### Detection of carnosine and acetyl-CoA content

Carnosine was determined by using a human carnosine ELISA kit (Dogesce, DG94186Q-48T) according to the manufacturer's instructions. Acetyl-CoA was determined by using an acetyl-CoA ELISA kit (Tianjin Zancheng, AE5076A-48T) according to the manufacturer's instructions.

### RNA extraction and quantitative real-time PCR

Total RNA was isolated with the use of TRIzol reagent (LABLEAD, R1000) and reverse transcribed with Superscript kit (HiScript^R^ II Q RT SuperMix for qPCR, R233-01). The initial denaturation step of PCR amplification was 95 ℃ for 5 minutes, followed by 40 cycles of 95 ℃ for 10 seconds and 60 ℃ for 30 seconds, then melting at 95 ℃ for 30 seconds and 60 ℃ for 30 seconds, and last at 95 ℃ for 30 seconds. The mean CT value of each gene was calculated, and the CT value of the target gene for each group minus the internal reference gene was the first Δ CT. The mean of the control group ΔCT was then calculated, and each of the control and experimental groups was used Δ CT minus control group Δ CT mean to obtain ΔΔ CT. Finally, 2^- ΔΔ CT^ was calculated. Gene expression was normalized against β-Actin or GAPDH.

### Western blot analysis

Cell lysates were prepared by using RIPA lysis buffer (MACGENE, MP015) supplemented with complete protease inhibitor cocktail. After the concentration was determined, protein samples were mixed with reducing buffer, boiled for 10 minutes, separated on Sodium dodecyl-sulfate polyacrylamide gel electrophoresis (SDS-PAGE), transferred to nitrocellulose filter membrane, blocked with 5% nonfat milk in Tris-buffered saline with Tween 20, and then incubated with primary antibodies at 4 ℃ overnight. The primary antibody including anti-CDH5 (Proteintech, 66804-1-Ig), anti-VIM (Bioss, bsm-33170M), anti-β-Actin (Proteintech, 66009-1-Ig), anti-ALDH2 (Proteintech, 15310-1-AP), anti-ALDH3A1 (ABclonal, A5502), anti-ALDH6A1 (Proteintech, 20452-1-AP), anti-DNMT1 (ABclonal, A15050), anti-Smad2/3 (ABclonal, A7536), and anti-pSmad2/3 (ABclonal, AP0548). Bound antibodies on the membrane were detected by secondary antibodies against rabbit IgG (ZSGB-BIO, ZB-2301) or mouse IgG (ZSGB-BIO, ZB-2305) and visualized by Odyssey infrared imaging system (LI-COR Biosciences) or Molecular Imager ChemiDoc XRS + System (Bio-Rad).

### Chromatin immunoprecipitation (ChIP)

Cells were washed once with PBS (room temperature), crosslinked with 1% formaldehyde in PBS for 10 min, rinsed twice with ice-cold PBS and resuspended with 300 μl of lysis bufer (1% SDS, 5 mmol/L EDTA, 50 mmol/L Tris-HCl (pH 8.1), and protease inhibitors), incubated on ice for 10 min, and sonicated for 3 times. 20% aliquot was saved as an input. The lysate was 1:10 diluted in dilution buffer (1% Triton X-100, 2 mmol/L EDTA, 150 mmol/L NaCl, 20 mmol/L Tris-HCl (pH 8.1), and protease inhibitors), and was incubated with antibody against DNMT1 for 6 h or overnight at 4 °C and then with 30 μl of protein A-G sepharose beads for another 2 hours. The sepharose beads were washed sequentially with buffer TSE I (0.1% SDS, 1% Triton X-100, 2 mmol/L EDTA, 20 mmol/L Tris-HCl, pH 8.1, 150 mmol/L NaCl), buffer TSE II (0.1% SDS, 1% Triton X-100, 2 mmol/L EDTA, 20 mmol/L Tris. HCl, pH 8.1, 500 mmol/L NaCl), buffer III (0.25 mol/L LiCl, 1% NP-40, 1% deoxycholate, 1 mmol/L EDTA, 10 mmol/L Tris. HCl, pH 8.1), and TE buffer. Crosslinks were stayed at 65 °C overnight. DNA was pured with DNA Pure-Spin Kit (Vigorous), and subjected for PCR amplification. Primer list is presented in Table S1.

### DNA isolation and methylation-specific polymerase chain reaction (MSP)

Genomic DNA was extracted from cells with DNA extraction buffers (50 μl TE buffer, 450 μl STE buffer,10 μl 20% SDS,10 μl protein K (10 mg/ml)) 39. Bisulfite modification of DNA (1 μg) was performed by using the EpiJET Bisulfite Conversion Kit (Thermo scientific, K1461) according to the manufacturer's instructions. DNA was purified with a DNA Pure-Spin Kit (Vigorous, N009). MethPrimer platform was used for designing bisulfite-conversion-based MSP primers for ALDH2, ALDH3A1 and ALDH6A1. Polymerase chain reaction (PCR) amplifications were carried out in a total volume of 25 μl by using Taq DNA Polymerase (GenStar, A012-B101). The initial denaturation step of PCR amplification was 95 ℃ for 5 minutes, followed by 43 cycles of 95 ℃ for 30 seconds and 52 ℃ for 30 seconds and 72 ℃ for 30 seconds, then maintaining at 72 ℃ for 5 minutes, and last at 12 ℃ for infinite. MSP products were separated on 2% agarose gels and visualized after Gel-Red (Beyotime Biotechnology, Shanghai) staining. Primers are presented in Table S1.

### Treatment of HUVECs with 5-aza-2'-deoxycytidine (5-Aza)

5-Aza was purchased from Selleck (S1200) and was diluted with DMSO to a storage concentration of 50 mmol/L for *in vitro* test. 5-Aza was added into the culture medium at a concentration of 10 mmol/L at 24 hours before applying PS or OS to the treated cells. 5-Aza (50 mmol/L) was dissolved in normal saline at a final concentration of 0.2 mg/mL for intraperitoneal injection in animals.

### Chemicals and reagents

TGF-β1 was purchased from Abbkine (PRP100190) and was diluted with sterile water to a storage concentration of 10 mg/mL. Carnosine was from SAITONG (A10437-5g) and was diluted with sterile water to a storage concentration of 500 mmol/mL. Sodium acetate was from Sigma-Aldrich (S5636) and was diluted with sterile water to a storage concentration of 4 mol/mL.

### Transient transfection with siRNA or plasmids

siRNA was transfected into HUVECs using Lipofectamine 2000 (Invitrogen, 12252-011) with either siALDH2, siALDH3A1, or siALDH6A1 (QingKe) at 100 nmol/L or scrambled control siRNA (QingKe) in OptiMEM (Invitrogen, 31985-088;). Six hours after transfection, the medium was replaced with normal culture medium. Forty-eight hours later, shear stress (either PS or OS) was applied to the transfected cells for an additional 24 hours.

To overexpress ALDHs, plasmids harboring the full-length sequences of ALDH2 (YouBio, NM_000690), ALDH3A1 (YouBio, NM_000691), or ALDH6A1 (YouBio, NM_005589) were transfected into EA. hy926 cells using Lipofectamine 2000 to achieve a higher transfection efficacy. Six hours after transfection, the medium was replaced with normal culture medium. Forty-eight hours later, shear stress (either PS or OS) was applied to the transfected cells for an additional 24 hours.

### Immunofluorescence staining

Tissues were first washed with PBS buffer and adventitia was removed carefully, and then they were fixed with 4% paraformaldehyde (PFA) and embedded in 20% sucrose solution before being frozen in TissueTek cutting medium (Sakura Finetek). 8 μm sections were processed for immunofluorescent analysis. The sections were further fixed with 4% PFA for 20 minutes. For immunostaining of attached cells, cells were fixed in 4% PFA for 20 minutes and permeabilized with 0.1% Triton X-100 (in PBS) for 5 minutes and rinsed for 3 times. Nonspecific binding was blocked by 4% BSA in PBS for 1 hour. Tissues/cells were incubated at 4 °C overnight in incubation buffer containing 4% BSA and the primary antibodies including anti-CDH5 (1:100), anti-Ki67 (Abcam, ab15580, 1:200), anti-F4/80 (BioLegend, 157309, 1:100), anti-SM22α (Proteintech, 10493-1-AP, 1:100), anti-ALDH2 (1:100), anti-ALDH3A1(1:100), anti-ALDH6A1 (1:100), and anti-pSmad2/3 (1:100). After being washed in PBS for 3 times, the specimens were incubated with Alexa Fluor 488-conjugated goat anti-rabbit/-mouse IgG (Abcam, ab150077/ab150113) or Alexa Fluor 555-conjugated goat anti-rabbit/-mouse IgG (Abcam, ab150078/ab150114) or Alexa Fluor® 647 goat anti-rabbit igG (Abcam, ab150083) for 1 hour at room temperature. The fluorescent signals were detected by fluorescence microscopy (Leica TCS SP8).

### Animals

All animal studies were performed in accordance with the approved protocol of the Animal Care and Use Committee of Peking University and approved by the Ethics Committee of Peking University Health Science Center (LA2019262). Dnmt1^flox/flox^ (B6.129S4-Dnmt1^tm2Jae^/Mmucd, MMRRC, stock number 014114-UCD) mice were obtained from the Jackson Laboratory. Dnmt1^flox/flox^ mice were maintained in a C57BL/6 background on a standard chow and crossed with vascular endothelial-cadherin Cre recombinase-positive mice (Cdh5(PAC)-Cre^ERT2^) to generate the Dnmt1^flox/flox^ Cdh5-Cre^ERT2+^ (Dnmt1^ECKO^) mice. Dnmt1^flox/flox^ Cdh5-Cre^ERT2-^ (Dnmt1^WT^) littermates were used as the controls. Rosa26-mTmG mice (JAX Stock No. 007676) ubiquitously express membrane-targeted tandem dimer Tomato (tdTomato) reporter protein from the R26 locus under the transcriptional regulation of Actb in the absence of cre recombinase. The mice express membrane-targeted enhanced green fluorescent protein (EGFP) upon Cre-mediated excision of the floxed stop signal cassette 40. Rosa26-mTmG mice were crossed to Tek-Cre (JAX Stock No.008863) mice to generate the EC- lineage tracing EndoTrack^EGFP^ mice. Mice were housed in specific pathogen-free cages, 12-hour light-dark cycle, controlled temperature and humidity, and had water and food ad libitum. Anesthetization was performed by using a small animal anesthesia machine (RWD, cat. no. R520) with isoflurane (RWD, cat. no. R510-22). Anesthesia was maintained by inhalation of 0.1% isoflurane through breathing mask during the operation. Euthanasia was performed by isoflurane overdose through breathing mask.

### AAV8-PCSK9 production and purification

AAV8-PCSK9 viruses were produced and purified as described previously 41. The gain-of-function murine PCSK9 mutant plasmid (pAAV/D377YmPCSK9) was a gift from Jacob Bentzon (Addgene plasmid # 58376). AAV-plasmids were cloned and propagated in the DH5αE. coli strain (Life Technologies) in medium with ampicillin. Shuttle plasmid pAAV-D377Y mPCSK9 was packaged into capsids AAV8, using helper plasmids phelper (providing the three adenoviral helper genes) and plasmid pAAV2/9 (providing rep and cap viral genes). The amplified AAV shuttle and helper plasmids were co-transfected into HEK293T cells by polyethyleneimine (PEI). A total of 420 μg of plasmid DNA (mixed in an equimolar ratio) 8 were used for 30×100-mm plates seeded with 1×10^7^ cells per plate. 72 hours post-transfection, cell culture media and transfected cells were harvested separately. The cell pellet was suspended in 1 ml of PBS and 10% sodium deoxychalate, and then 50 μl of DNAase were added, sonicated with a 15-45 seconds cycle for 10 times. Cell debris was pelleted by spinning at 12,000g for 30 minutes at 4 ℃. The lysate and supernatant were sequentially filted through 0.45 μm filter and 0.22 μm filter. 40% polyethylene glycol (PEG) in 2.5 mol/L NaCl was added to the liquid to a final concentration of 8% and precipitated for 4 hours. The PEG-precipitated viruses were pelleted by centrifugation at 2,500g for 20 minutes at 4 ℃. The precipitations were washed by with 8% PEG, and then 20 ml of PBS with 4% sucrose was added to resuspend the pellets. The mixture was centrifuged at 2,500g for 15 minutes at 4 ℃ to obtain the supernatant, which was then dialyzed through an Amicon Ultra 100,000 MWCO concentration unit (Merck-Millipore) to acquire purified virus. The viruses were aliquoted and stored at -80 ℃.

### Blood pressure measurement in mice

Blood pressures were recorded by using a mouse & rat tail-cuff blood pressure system (Kent, cat. no. coda8). Mice were placed in the restraint corridor and allowed at least 10 minutes of acclimation. The area was warmed with a heating pad and a quiet, dark environment was maintained to ensure reliable measurements within the parameters of this technology. The mice underwent 3 consecutive days of training sessions from 1 to 5 PM each day to become accustomed to the tail-cuff procedure. Five measurements were daily performed on each mouse and the blood pressure was the mean value of five successful measurements.

### Detection of mice blood triglyceride (TG) and total cholesterol (TC)

Mice blood TG was determined with a triglyceride assay kit (Nanjing Jiancheng, cat. no. A110-1-1) according to the manufacturer's instructions. Mice blood TC was determined with a total cholesterol assay kit (Nanjing Jiancheng, cat. no. A11C1-1-1) according to the manufacturer's instructions.

### Partial carotid ligation in mouse

Partial ligation of carotid artery in mice was performed as we previously reported 19, 42. Briefly, the left carotid bifurcation of mice was exposed following a neck incision. Three branches (external carotid, internal carotid, and occipital) of the left carotid artery were ligated with a 6-0 silk suture, and the superior thyroid artery was left intact. The right carotid arteries served as controls. Disturbed flow generated in the left carotid arteries.

### Disturbed flow-accelerated atherosclerotic mouse model

Dnmt1^ECKO^ and Dnmt1^WT^ mice (8 weeks) were subjected to tamoxifen (dissolved in corn oil Sigma-C8267) injection via the intraperitoneal route (1.5 mg daily per mouse for 7 days), and then partial carotid artery ligation was performed. The mice were then subjected to AAV8-PCSK9 virus injection via the tail vein (once) followed by a high cholesterol diet (Research Diets, D12108C, High fat rodent diet with 1.25% cholesterol) to induce hyperlipidemia 43, 44. The mice were sacrificed after 6 weeks of high cholesterol diet.

### Hematoxylin and eosin (H&E) staining

After rehydration, 8-μm frozen sections were stained with hematoxylin solution for 3 minutes followed by 2 dips in 1% acid ethanol (1% HCl in 70% ethanol) and then rinsed in running water. Then the sections were stained with eosin solution for 5 minutes followed by dehydration with graded alcohol (70%, 80%, 90%, 95%, 100%) and clearing in xylene.

### Oil Red O Staining

Vascular frozen sections were washed 3 times with PBS, fixed in 4% PFA for 10 minutes, and then washed with double distilled water for 3 times. The sections were immersed in 60% isopropanol solution for 10 minutes, stained with Oil Red O for 30 minutes, washed several times in 60% isopropanol solution, and finally rinsed with double distilled water. Glycerol gelatin was used for sealing.

### Analysis of high-throughput datasets

The original microarray data from Dunn J et al.'s paper 15 was downloaded from the GEO database (GEO GSE56143). Fold-change was calculated by the mean ratio of the sequencing values between the two groups. A total of 944 genes from the left carotid artery over the right carotid artery (fold-change ≤ 0.75) in the DMSO-treated group and the 5-Aza-treated left carotid artery over the DMSO-treated left carotid artery (fold-change ≥ 1.25) were obtained. KEGG analysis of these 944 genes was carried out by using DAVID Bioinformatics Resources.

The 1,365 differentially expressed genes of OS versus PS (|log2 fold change| ≥ 0.33) from our own RNA-sequencing data (BioProject ID PRJNA772796) 43 obtained from HUVECs with 6-hours-exposure to PS or OS were analyzed by using ChEA3 to generate the predicted enrichment of transcription factors.

The 458 overlapping genes between Dunn et al.'s microarray dataset (genes differentially expressed between the ligated left carotid artery and the unligated right carotid artery) and our laboratory's RNA-seq dataset (genes differentially expressed between OS and PS) were obtained. The 458 overlapping genes were analyzed by using ChEA3 to generate the predicted enrichment of transcription factors.

### Generation of graphical abstract

Graphical abstract was drawn by using the Figdraw platform.

### Statistical analysis

Data are presented as mean ± SEM of the mean from at least three independent experiments as indicated. For in-vitro experiments, as each experimental data set is an average of a large number of cultured cells, we assumed the data was normally distributed based on the central limit theorem. For in-vivo experiments, the n value represents independent repeats or the numbers of animals. All analysis was performed using GraphPad Prism version 9.0.0. Normality of data distribution was tested with a D'Agostino-Pearson or Shapiro-Wilk test. For normally distributed data, differences between treatment groups were determined using unpaired t-test for two groups of data and one-way or two-way ANOVA for multiple groups of data. Statistical significance among multiple groups was determined by post hoc analysis (Tukey's multiple comparisons test or Dunnett's multiple comparisons test or Newman-Keuls multiple comparisons test). Values of *P* < 0.05 were considered statistically significant.

## Results

### Inhibition of DNMTs with 5-Aza suppressed the disturbed flow-induced EndoMT

**Figure 1:**
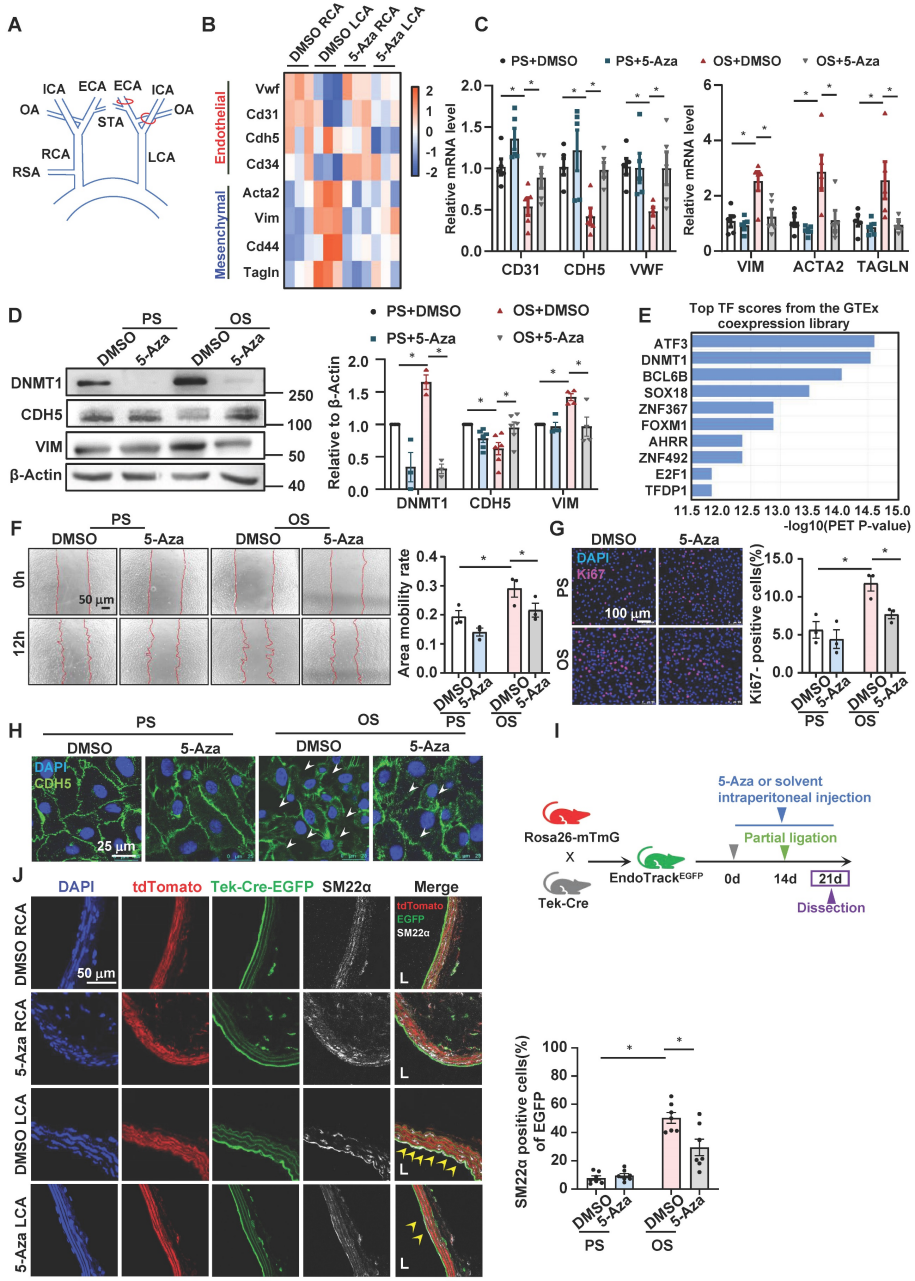
** Inhibition of DNMTs with 5-Aza suppressed the disturbed flow-induced EndoMT. (A)** Schematic diagram of the partial carotid ligation surgery. ECA: external carotid artery; ICA: internal carotid artery; OA: occipital artery; RSA: right subclavian artery; STA: superior thyroid artery. LCA: Left common carotid artery; RCA: Right common carotid artery. **(B)** Heatmaps of the marker genes of endothelial cells and mesenchymal cells in the DMSO RCA group, DMSO LCA group, 5-Aza RCA group, and 5-Aza LCA group. LCA: Left common carotid artery; RCA: Right common carotid artery. **(C**-**D)** HUVECs were pretreated with DMSO or 5-Aza (10 μmol/L) for 24 hours, exposed to PS (12 ± 4 dynes/cm^2^) or OS (0 ± 4 dynes/cm^2^) for 24 hours, and the protein and mRNA expressions of EndoMT-related genes were assayed by quantitative RT-PCR **(C)** and Western blotting** (D)**. Data were presented as mean ± SEM. *P < 0.05 by two-way ANOVA followed by Tukey's multiple comparisons test, n = 3-6. **(E)** Enrichment of transcription factors of differentially expressed genes in the transcriptome of HUVECs exposed to PS/OS for 6 hours. **(F)** HUVECs were pretreated with DMSO or 5-Aza (10 μmol/L) for 24 hours, exposed to PS/OS for 6 hours, and the cell migration was detected by a scratch wound assay. Data were presented as mean ± SEM. *P < 0.05 by two-way ANOVA followed by Newman-Keuls multiple comparisons test, n = 3. **(G-H)** HUVECs were pretreated with DMSO or 5-Aza (10 μmol/L) for 24 hours, exposed to PS/OS for 24 hours, and immunofluorescence staining was carried out with anti-Ki67 (red) **(G)** and anti-CDH5 (green) **(H)** antibodies (DAPI nuclear staining was in blue). **(G)** Each dot represents the average value obtained from at least 5 fields in each experiment. Data were presented as mean ± SEM. *P < 0.05 by two-way ANOVA followed by Newman-Keuls multiple comparisons test, n = 3. **(I)** Schematic diagram of the experimental design in the EC-linage tracing EndoTrack^EGFP^ mice. **(J)** The EndoTrack^EGFP^ mice were subjected to partial carotid ligation and 5-Aza/DMSO administration, and the common carotid arteries (both LCA and RCA) were collected for assessing EndoMT. Tek-Cre-EGFP (green) fluorescence indicates ECs and the autofluorescent elastic lamina. TdTomato (red) fluorescence indicates the rest of the vascular wall cells. Mesenchymal marker is illustrated by SM22α immunofluorescence staining (white). DAPI (blue) indicates the nuclei. EGFP^+^SM22α^+^ cells (arrowheads) represent endothelial-derived cells expressing mesenchymal marker. L: lumen. Data were presented as mean ± SEM. Each dot represents the average value obtained from three distinct regions (upper, middle, and lower) of the common carotid arteries of mice. *P < 0.05 by two-way ANOVA followed by Tukey's multiple comparisons test, n = 7.

We analyzed the microarray data (GEO GSE56143) from Dunn J et al.'s prior work 15, where C57BL/6 mice were subjected to intraperitoneal injection with a potent DNMT inhibitor, 5-Aza 19, 20, 45, 46, or control solvent, and partial ligation of their left carotid artery (LCA) (Figure 1A) to induce disturbed flow in LCA but not the right carotid artery (RCA), and endothelia from both carotid arteries were collected to apply for transcriptional profiling. We looked for the expression of endothelial vs. mesenchymal marker genes. Compared with the solvent control of the RCA, LCA exhibited a reduced expression of endothelial markers (i.e., Cd31, Cd34, Vwf and Cdh5) and a promoted expression of mesenchymal markers (i.e., Tagln, Cd44, Vim and Acta2) (Figure 1B), indicative of EndoMT. These effects were abolished by administration with 5-Aza (Figure 1B). This analysis suggests a role of DNMTs in mediating the flow-induced EndoMT. To further test this, we measured the expression of the marker genes by quantitative RT-PCR and Western blotting in cultured HUVECs that had been subjected to 5-Aza (10 μmol/L, 24 hours) or DMSO treatments followed by mechanical loading with PS (12 ± 4 dynes/cm^2^) or OS (0.5 ± 4 dynes/cm^2^) for another 24 hours. The results showed that compared with the PS treated with DMSO, OS decreased the expression of endothelial markers whereas increased the expression of mesenchymal markers, and that this influence could be abrogated by 5-Aza (Figure 1C-D). Next, we studied our own RNA-sequencing data (BioProject ID PRJNA772796) 43 obtained from HUVECs with 6-hours-exposure to PS or OS. Transcription factor enrichment analysis by ChEA3 47 indicated that DNMT1 ranked up to the 2^nd^ of the top transcription factors associated with the differentially expressed genes (Figure 1E), hinting at an importance of DNMT1 in mediating the shear-induced transcriptional changes. In addition, transcriptional factor enrichment analysis of the cross-overlapping genes between Dunn J et al.'s microarray data and genes differentially expressed between OS and PS in our own RNA-sequencing data showed that DNMT1 ranks 15^th^ (Figure S1). The *in vitro* results confirmed that 5-Aza can inhibit the increase in DNMT1 expression caused by OS (Figure 1D).To explore the functional role of DNMTs during EndoMT that is characterized by enhanced cell migration and proliferation, as well as loss of the endothelial intercellular junction 9, we first performed scratch wound healing and Ki67 immunofluorescence staining experiments and found that treatment of ECs with 5-Aza suppressed the OS-induced cell migration and proliferation (Figure 1F-G). Immunofluorescence staining of the endothelial junctional adhesion protein, CDH5, then indicated that while the EC monolayer under PS had complete and tight connections, the cells under OS exhibited junction interruption; the differences between OS and PS could be reduced by 5-Aza (Figure 1H). To further validate the role of DNMTs in EndoMT *in vivo*, we used the Tek-Cre and Rosa26-mTmG hybridized transgenic mice EndoTrack^EGFP^ equipping with an endothelial lineage tracing system. TdTomato fluorescence expression is widespread in cells/tissues from the mice; however, Tek-Cre recombinase expressing cells (specifically, ECs here) have cell membrane-localized EGFP fluorescence replacing the red fluorescence. EndoTrack^EGFP^ mice were subjected to intraperitoneal injection with 5-Aza (0.2 mg/kg, 6 consecutive days of injection and 1 day of rest as intervals for 3 weeks) to inhibit DNMTs or with DMSO in normal saline, and a surgery of partial ligation of their carotid arteries performed on the last week to create disturbed flow in LCA but not RCA (Figure 1I). Both RCA and LCA were collected and prepared for sections. In RCA with/without 5-Aza treatment, EGFP-expressing ECs lined up the inner surface of the lumen, and they were barely observed for expression of mesenchymal marker SM22α visualized by immunofluorescent staining (the white signals) (Figure 1J). The rest cells in the sections expressing red tdTomato fluorescence were mainly SMCs that were SM22α-positive (Figure 1J). In LCA with DMSO, a strong expression of SM22α was found in the EGFP-expressing ECs; in comparison, in LCA with 5-Aza, the white SM22α signals in the EC-lineaging cells were largely eliminated (Figure 1J). These findings from the lineage tracing model validated the conclusion that DNMTs mediate the disturbed flow-induced EndoMT.

### 5-Aza ameliorated the downregulation of ALDH expression and β-alanine biosynthesis by OS

**Figure 2:**
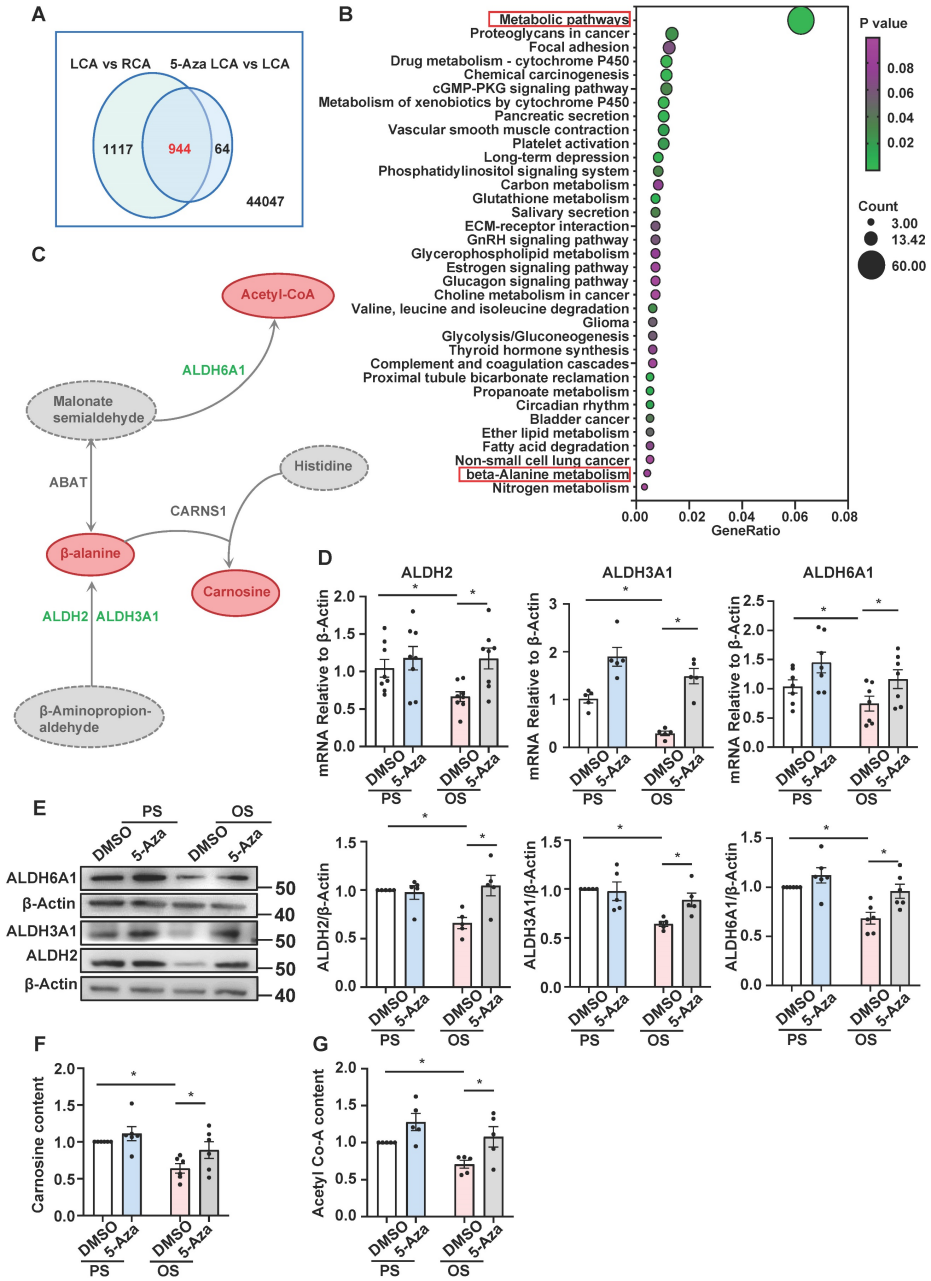
**5-Aza ameliorated the downregulation of ALDH expression and β-alanine biosynthesis by OS. (A)** Webster's chart of gene expression difference. **(B)** 944 probes selected from** (A)** were subjected to KEGG pathway enrichment analysis. 35 pathways were presented. **(C)** Schematic diagram illustrating the role of ALDH2, ALDH3A1, and ALDH6A1 in β-alanine metabolic pathway. **(D-E)** HUVECs were pretreated with DMSO or 5-Aza (10 μmol/L) for 24 hours, exposed to PS or OS for 24 hours, and the protein and mRNA expressions of ALDH2, ALDH3A1 and ALDH6A1 were assayed by quantitative RT-PCR** (D)** and Western blotting** (E)**. Data were presented as mean ± SEM. *P < 0.05 by two-way ANOVA followed by Tukey's multiple comparisons test, n = 5-8. **(F-G)** HUVECs were pretreated with DMSO or 5-Aza (10 μmol/L) for 24 hours, exposed to PS or OS for 24 hours, carnosine content **(F)** and acetyl-CoA content **(G)** were measured by ELISA kit. Data were presented as mean ± SEM. *P < 0.05 by two-way ANOVA followed by Newman-Keuls multiple comparisons test, n = 5 or 6.

Further analysis of Dunn J et al.'s microarray data 15 indicated that 2,061 genes were downregulated in LCA vs. RCA (fold-change ≤ 0.75) and that in 944 of which the downregulation could be inhibited by 5-Aza (5-Aza vs. DMSO in LCA, fold-change ≥ 1.25) (Figure 2A), echoing a role of DNMTs in mediating gene silencing.

Kyoto Encyclopedia of Genes and Genomes (KEGG) pathway analysis of the 944 genes revealed that they were enriched in 35 pathways, a large number of which belong to metabolic pathways such as carbon metabolism, glutathione metabolism, glycerophospholipid metabolism, glycolysis and gluconeogenesis, propanoate metabolism, ether lipid metabolism, β-alanine metabolism, and nitrogen metabolism (Figure 2B). In the 60 genes of metabolic pathways, ABAT, ALDH2, ALDH3A1, and ALDH6A1 were indicated by protein-protein interaction analysis (provided by the STRING online server) to be tightly connected (Figure S2). Among them, ALDH2, ALDH3A1, and ALDH6A1 are subunits of the ALDH family proteins and play different but important roles in the biosynthesis and metabolism of β-alanine (Figure 2C). To deeply look into these genes, we measured their expression in 5-Aza-treated (24 hours, 10 μmol/L) HUVECs followed by exposure to 24-hours-PS or OS. Quantitative RT-PCR and Western blotting assays indicated that compared with PS/DMSO, OS inhibited both the mRNA and protein levels of ALDH2, ALDH3A1, and ALDH6A1, and that the inhibitory effect could be abolished by 5-Aza (Figure 2D-E), consistent with the microarray results (Table S2). Due to the technique difficulty, we were not able to measure the β-alanine levels in cells. Given that β-alanine is the rate-limiting precursor of carnosine synthesis, we measured the carnosine levels instead. As ALDH6A1 mediates the biosynthesis of acetyl-CoA, we also assessed the acetyl-CoA levels the cells with the above treatments. We found that the cellular biosynthesis of carnosine and acetyl-CoA was reduced by OS, and that the reduction could be blocked by treatment with 5-Aza (Figure 2F-G), in line with the shear-regulated and DNMT-mediated transcriptional regulation of the ALDH genes. Altogether, results from these experiments demonstrate that the expression of ALDH2, ALDH3A1, and ALDH6A1, as well as the β-alanine metabolism are changed by shear stress and the changes may be mediated by DNMTs.

### 5-Aza ameliorated the downregulation of ALDH expression by disturbed flow in mice

**Figure 3:**
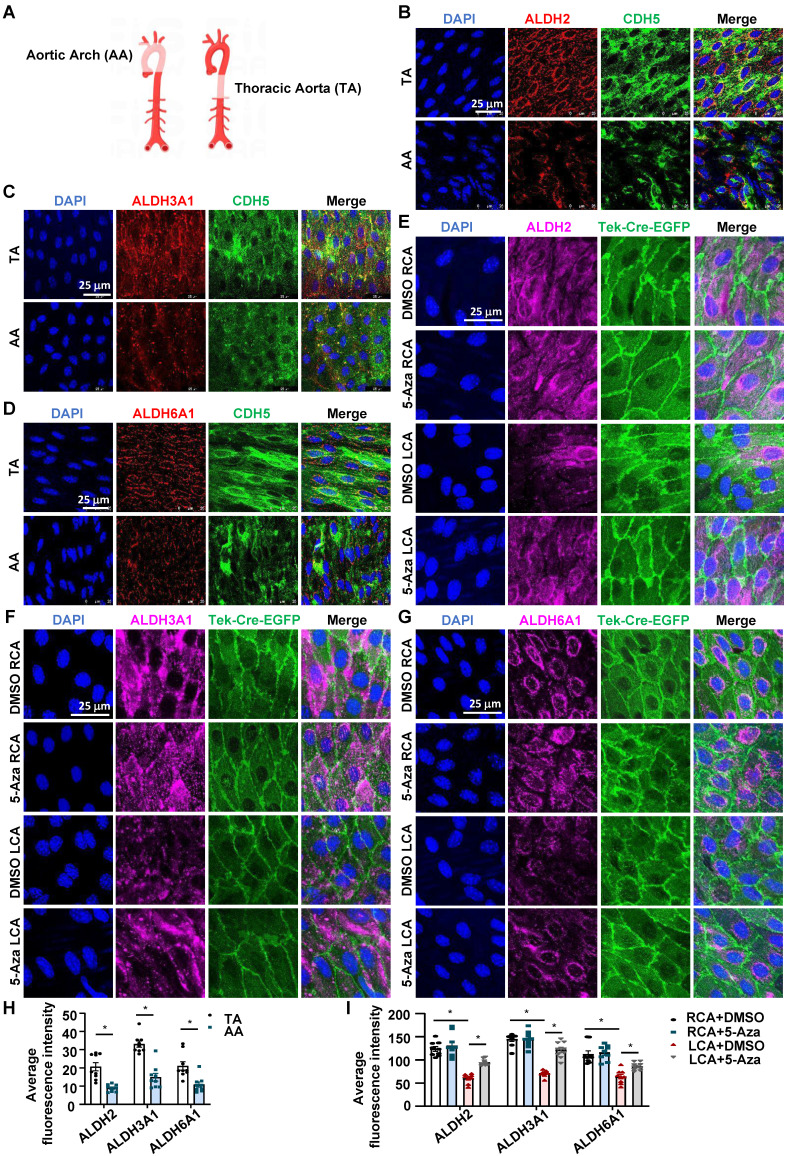
** 5-Aza ameliorated the downregulation of ALDH expression by disturbed flow in mice. (A)** Schematic diagram of TA (Thoracic aorta) and AA (Aortic arch). Schematic diagram was drawn by using the Figdraw platform.** (B-D)** En face immunofluorescence staining with anti-ALDH2 (red), anti-ALDH3A1 (red), anti-ALDH6A1 (red) and anti-CDH5 (green) antibodies in the endothelia from TA and AA. DAPI (blue) indicates the nuclei. **(E-G)** En face immunofluorescence staining with anti-ALDH2 (magenta), anti-ALDH3A1 (magenta), anti-ALDH6A1 (magenta) antibodies in the endothelia from LCA and RCA. DAPI (blue) indicates the nuclei. Tek-Cre-EGFP (green) fluorescence indicates ECs. LCA: Left common carotid artery; RCA: Right common carotid artery. **(H-I)** Quantification of immunofluorescence intensity was conducted by ImageJ software; n = 9 sections from 3 mice. Data were presented as mean ± SEM. *P < 0.05 by conditional hierarchical linear mixed effect model.

To explore the *in vivo* relevance of the *in vitro* effects of DNMT inhibition on flow-regulated ALDH expression, we assessed the endothelial expression of ALDH2, ALDH3A1, and ALDH6A1 in mice by using en face immunofluorescent staining, a technique allowing researchers to observe the entire surface of the endothelium clearly and to compare the expression patterns of a given protein in regions under different fluid shear stress 48. In mammals such as humans and mice, the inner curvature of the aortic arch (AA) is a naturally disturbed flow region, whereas the long straight part of the thoracic aorta (TA) is usually exposed to laminar flow 4 (Figure 3A). En face immunofluorescent staining of the endothelia in TA and AA revealed that compared with TA, AA exhibited a lower expression of ALDH2, ALDH3A1, and ALDH6A1 (Figure 3B-D and Figure 3H). Staining of the endothelial marker CDH5 illustrated a correct type of cells that were assessed. We also tested the endothelial expression of those genes in EndoTrack^EGFP^ mice that were subjected to partial carotid ligation and 5-Aza injection. As expected, compared with the expression of ALDHs in the unligated RCA, the expression of ALDH2, ALDH3A1and ALDH6A1 in the ligated LCA was markedly reduced; these reductions were suppressed by 5-Aza (Figure 3E-G and Figure 3I). These observations were consistent with *in vitro* findings.

### ALDH2, ALDH3A1, and ALDH6A1 were involved in the OS-induced EndoMT

**Figure 4:**
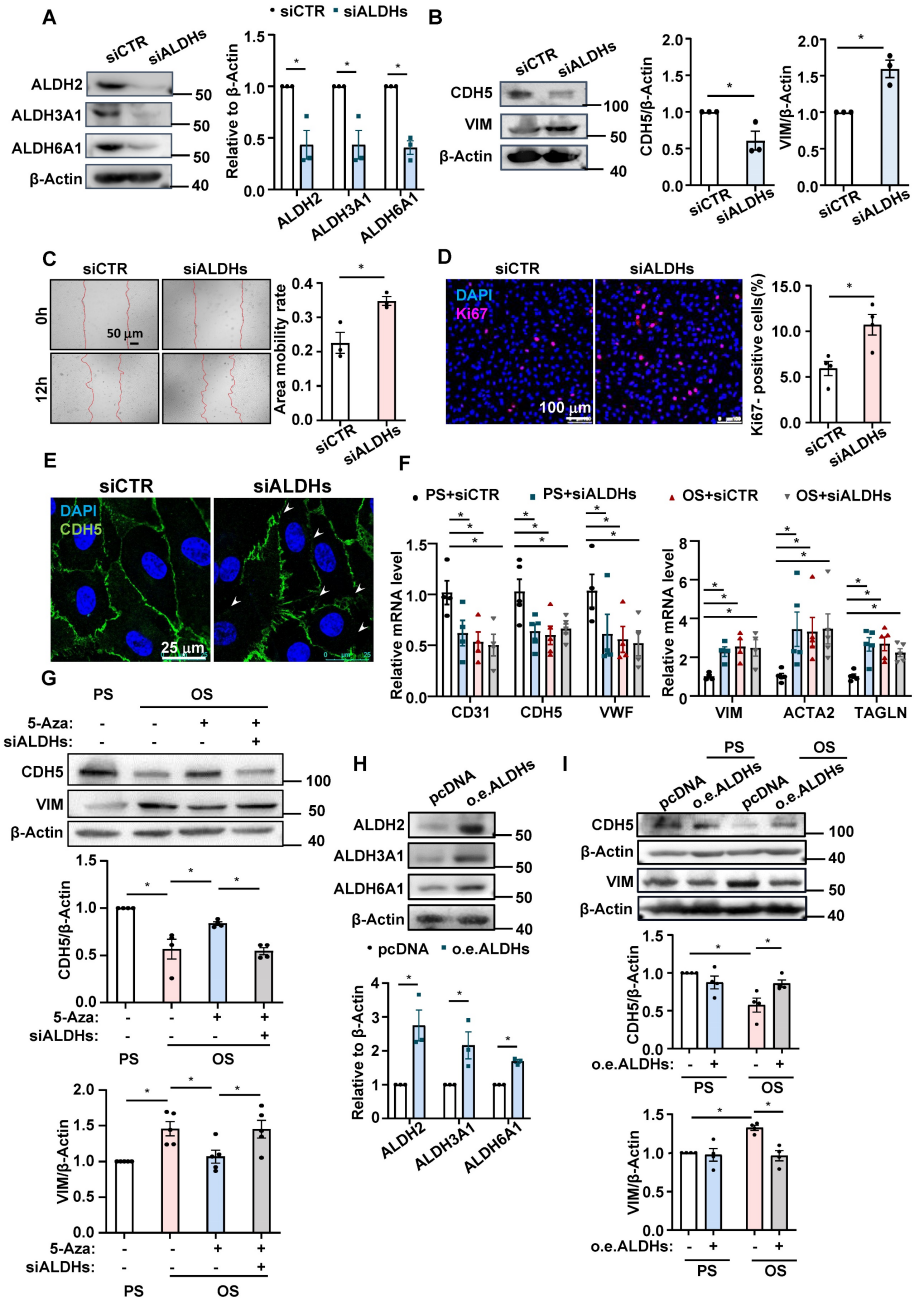
** ALDH2, ALDH3A1, and ALDH6A1 were involved in the OS-induced EndoMT. (A-B)** HUVECs were transfected with ALDH2-specific siRNA (siALDH2), ALDH3A1-specific siRNA (siALDH3A1) and ALDH6A1-specific siRNA (siALDH6A1) or the control siRNA (siCTR). Western blotting assay was performed to measure the expressions of ALDH2, ALDH3A1, ALDH6A1, CDH5 and VIM. Data were presented as mean ± SEM. *P < 0.05 by Unpaired t test, n = 3. **(C)** HUVECs were transfected with siALDH2, siALDH3A1 and siALDH6A1 or siCTR. Cell migration was measured by a scratch wound test. Data were presented as mean ± SEM. *P < 0.05 by Unpaired t test, n = 3. **(D-E)** HUVECs were transfected with siALDH2, siALDH3A1 and siALDH6A1 or siCTR. Immunofluorescence staining was carried out with anti-Ki67 (red) **(D)** and anti-CDH5 (green) **(E)** antibodies, DAPI nuclear staining was in blue. Data were presented as mean ± SEM. Each dot represents the average value obtained from at least 5 fields in each experiment. *P < 0.05 by Unpaired t test, n = 4. **(F)** HUVECs were transfected with siALDH2, siALDH3A1 and siALDH6A1 or siCTR, and then exposed to PS or OS for 24 hours, quantitative RT-PCR was used to measure the expression of CD31, CDH5, VWF, VIM, ACTA2 and TAGLN. Data were presented as mean ± SEM. *P < 0.05 by two-way ANOVA followed by Dunnett's multiple comparisons test, n = 4, 5. **(G)** HUVECs were pretreated with DMSO or 5-Aza (10 μmol/L) for 24 hours or transfected with siALDH2, siALDH3A1 and siALDH6A1 or siCTR, and then exposed to PS or OS for 24 hours, Western blotting to measure the expressions of CDH5 and VIM. Data were presented as mean ± SEM. *P < 0.05 by two-way ANOVA followed by Tukey's multiple comparisons test, n = 4 or 5.** (H)** EA. hy926 were transfected with the control vector (pcDNA) or plasmid expressing ALDH2, ALDH3A1 and ALDH6A1, Western blotting was used to measure the expressions of ALDH2, ALDH3A1 and ALDH6A1. Data were presented as mean ± SEM. *P < 0.05 by Unpaired t test, n = 3. **(I)** EA. hy926 were transfected with the control vector (pcDNA) or plasmid expressing ALDH2, ALDH3A1 and ALDH6A1, and then exposed to PS or OS for 24 hours, Western blotting was used to measure the expressions of CDH5 and VIM. Data were presented as mean ± SEM. *P < 0.05 by two-way ANOVA followed by Tukey's multiple comparisons test, n = 4.

To look for the link between ALDHs and EndoMT, we performed loss-of-function study of ALDH2, ALDH3A1, and ALDH6A1 by employing siRNA-mediated gene silencing. Knocking down the three genes in HUVECs individually or simultaneously with siRNA (Figure 4A and Figure S3) decreased the expression of endothelial marker CDH5 while increased the expression of mesenchymal marker VIM (Figure 4B and Figure S4), suggestive of a potential role of these genes in modulating EndoMT.

Given that disturbed flow and OS inhibited the expression of all the three genes, we manipulated them altogether in the subsequent study. Functional tests indicated that in a static status, knockdown of ALDHs including ALDH2, ALDH3A1, and ALDH6A1 promoted endothelial migration and proliferation and disrupted the intercellular junctions (Figure 4C-E). In HUVECs subjected to shear flow, compared with cells in PS with a control transfection, knockdown of ALDHs in PS caused decreases of the mRNA levels of the endothelial markers CD31, CDH5, and VWF and increases of the mRNA levels of the mesenchymal markers VIM, ACTA2, and TAGLN, mimicking the effects of OS on the marker gene expression (Figure 4F), suggesting that insufficient expression of ALDHs may lead to EndoMT and the impact is similar to the action of OS. Moreover, assessing the protein levels of the EndoMT marker genes with Western blotting showed that OS decreased the CDH5 expression while increased the VIM expression, and that treatment of the cells with 5-Aza could diminish such influence; once cells in an OS status had been pre-transfected with siALDHs, the protective effect of 5-Aza against EndoMT was abolished (Figure 4G). Further evidence from the gain-of-function study of ALDHs in an EA. hy926 cell line by using plasmid-mediated overexpression indicated that (Figure 4H), in cells subjected to shear flow, the OS-induced changes in the protein levels of CDH5 and VIM were compromised by ALDH overexpression (Figure 4I). Taken together, these findings demonstrate an involvement of ALDH2, ALDH3A1, and ALDH6A1 in the OS-induced EndoMT.

### Roles of DNMT-β-alanine metabolism-TGF-β signaling in the shear-modulated EndoMT

**Figure 5:**
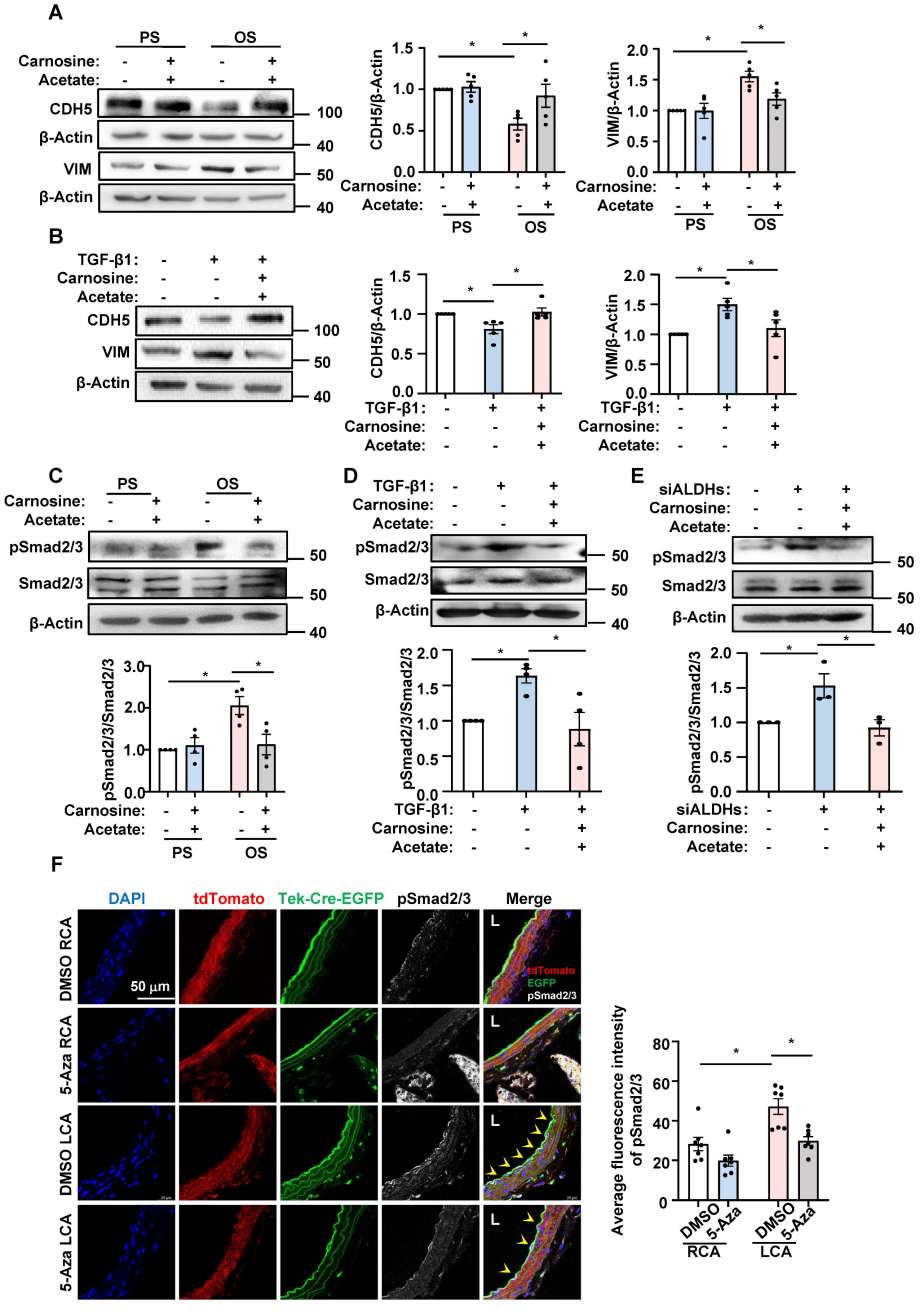
** Roles of DNMT-β-alanine metabolism-TGF-β signaling in the shear-modulated EndoMT. (A and C)** HUVECs were pretreated with sterile water or carnosine (500 µmol/L) and acetate (4 mmol/L) for 24 hours, exposed to PS or OS for 24 hours, and Western blotting was used to measure the expressions of CDH5, VIM, pSmad2/3 and Smad2/3. Data were presented as mean ± SEM. *P < 0.05 by two-way ANOVA followed by Tukey's multiple comparisons test, n = 4 or 5. **(B and D)** HUVECs were treated with TGF- β1 (10 ng/mL) for 3 days, and then carnosine (500 µmol/L) and acetate (4 mmol/L) or sterile water were added to the culture medium on the third day, while TGF- β1 continues to be treated, 24 hours later, the expression of CDH5, VIM, pSmad2/3 and Smad2/3 were measured by Western blotting. Data were presented as mean ± SEM. *P < 0.05 by one-way ANOVA followed by Tukey's multiple comparisons test, n = 4 or 5. **(E)** HUVECs were transfected with siALDH2, siALDH3A1 and siALDH6A1 or siCTR; 48 hours later, carnosine (500 µmol/L) and acetate (4 mmol/L) or sterile water were added to the culture medium. 24 hours later, western blotting was used to measure the expressions of pSmad2/3 and Smad2/3. Data were presented as mean ± SEM. *P < 0.05 one-way ANOVA followed by Tukey's multiple comparisons test, n = 3. **(F)** The EndoTrack^EGFP^ mice were subjected to partial carotid ligation and 5-Aza/DMSO administration, and the common carotid arteries (both LCA and RCA) were collected. Tek-Cre-EGFP (green) fluorescence indicates ECs and the autofluorescent elastic lamina. Immunofluorescence staining with anti-pSmad2/3 (white), DAPI (blue) indicates the nuclei. EGFP^+^ pSmad2/3^+^ cells were represented by arrowheads. L: lumen. Data were presented as mean ± SEM. Each dot represents the average value obtained from three distinct regions (upper, middle, and lower) of the common carotid arteries of mice. *P < 0.05 by two-way ANOVA followed by Tukey's multiple comparisons test, n = 7.

We next sought to demonstrate a role for β-alanine metabolism in EndoMT and to determine the mechanistic basis by which the β-alanine metabolites exert the action. Considering that the availability of β-alanine limits the production of carnosine, and that acetate can directly increase the acetyl-CoA level through the action of acetyl-CoA synthetase (ACSS2) 49, we supplemented HUVECs with carnosine (500 μmol/L) and acetate (4 mmol/L) and then assayed the OS-or TGF-β1-induced EndoMT. The results showed that combined supplementation of the cells with carnosine and acetate reversed the expressional changes of CDH5 and VIM caused by OS or TGF-β1 stimulation at the protein level (Figure 5A-B). Given that supplementation of mouse lung ECs or human mesangial cells with carnosine and/or acetate had been shown to inhibit the TGF-β1-induced phosphorylation of Smad2 25, 27, and that Smad2 and Smad3 are direct mediators of TGF-β signaling 50, we assessed the contribution of the β-alanine metabolites to the OS- or TGF-β1-stimulated phosphorylation of Smad2 and Smad3 (pSmad2/3). We found that combined supplementation of HUVECs with carnosine and acetate did suppress the OS- or TGF-β1-induced increase of pSmad2/3 without affecting the basal expression level of the total Smad2/3 (Figure 5C-D), supporting a mechanistic concept that a fall in carnosine and acetyl-CoA levels may act as a trigger for the activation of Smad2/3 that leads to EndoMT. Furthermore, the increased level of pSmad2/3 caused by knockdown of ALDH2, ALDH3A1, and ALDH6A1 could also be suppressed by supplementation with carnosine and acetate (Figure 5E), demonstrative of a role of these enzymes in mediating Smad2/3 activation. Snai1, Snai2, and Twist1 are well-known key transcription factors involved in EndoMT and they are activated by Smad2/3. We demonstrated that carnosine and acetyl-CoA inhibited the increased expression of Snai1, Snai2 and Twist1 caused by OS, TGF-β1 and ALDH2/3A1/6A1 knockdown (Figure S5-S7). In EndoTrack^EGFP^ mice that were subjected to partial carotid ligation and 5-Aza of control injection, a pronounced endothelial expression (indicated by Tek-Cre-EGFP) of pSmad2/3 (indicated by white signals) was observed in control mice in LCA where disturbed flow was experimentally generated; the pSmad2/3 level was markedly reduced in LCA upon administration with 5-Aza (Figure 5F). The results from the *in vitro* and *in vivo* studies have bridged the gap between DNMTs and EndoMT and suggested a role of DNMT-regulated β-alanine metabolism in mediating Smad2/3 activation.

### OS induced methylation for the promoter regions of ALDH2, ALDH3A1, and ALDH6A1 through DNMT1

In search of how DNMTs mediate the flow-regulated ALDH expression, we measured the methylation status for the promoter regions of ALDH2, ALDH3A1, and ALDH6A1 in HUVECs with PS vs.

**Figure 6:**
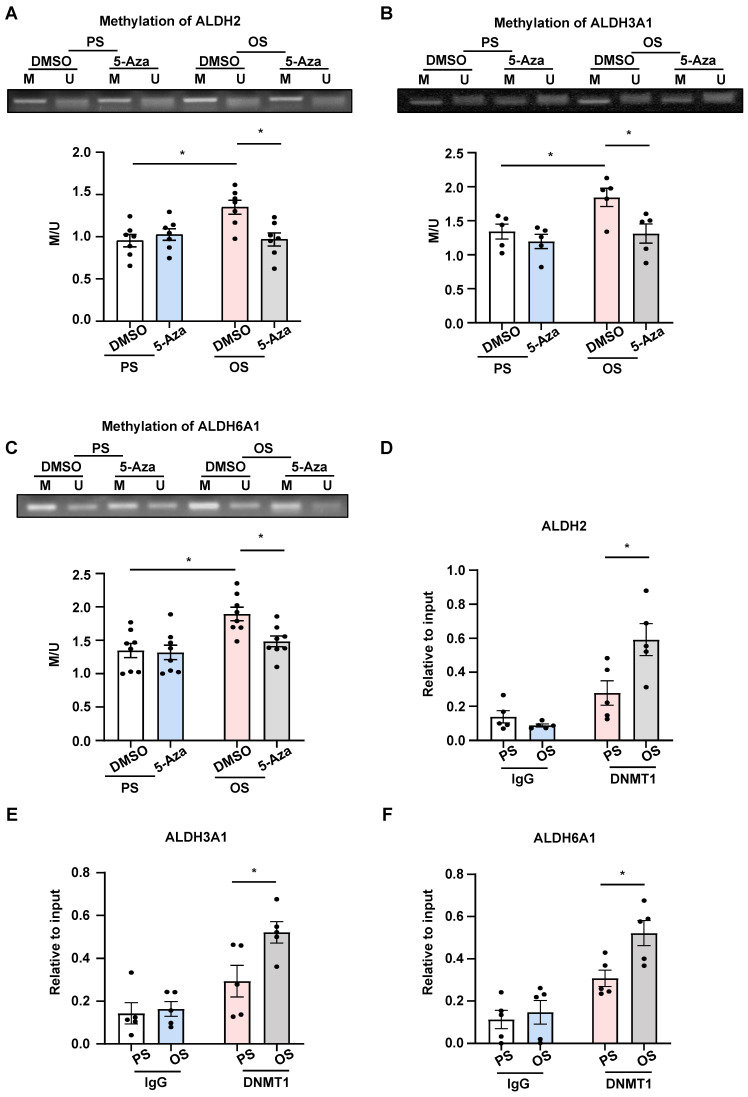
** OS induced methylation for the promoter regions of ALDH2, ALDH3A1, and ALDH6A1 through DNMT1. (A-C)** HUVECs were pretreated with DMSO or 5-Aza (10 μmol/L) for 24 hours, exposed to PS or OS for 24 hours, and MSP was used to measure the methylation level of ALDH2, ALDH3A1 and ALDH6A1 promoter regions. Data were presented as mean ± SEM. *P < 0.05 by two-way ANOVA followed by Tukey's multiple comparisons test, n = 5 or 7 or 8. **(D-F)** HUVECs were exposed to PS or OS for 24 hours and ChIP was used to measure the binding of DNMT1 to the ALDH2, ALDH3A1 and ALDH6A1 promoter regions. Data were presented as mean ± SEM. *P < 0.05 by two-way ANOVA followed by Tukey's multiple comparisons test, n = 5.

OS exposure and 5-Aza/DMSO treatment, as DNMT-mediated promoter hypermethylation is best known for their role in gene silencing 14, 15. An MSP method was used for analysis of the status of cytosine methylation in a cytosine-guanine dinucleotide at specific DNA loci 39. Using primer sets spanning the indicated loci at the promoter regions of the three genes (Figure S8), we found that application of OS to HUVECs induced hypermethylation of these ALDHs, and that administration with 5-Aza reversed the hypermethylation and took the status back to the basal (under PS) levels (Figure 6A-C). Given that we have previously reported an upregulation of DNMT1 in HUVECs upon OS exposure 20 and that here we found DNMT1 ranking the top 2 transcription factors associated with the differentially expressed genes generated from the transcriptome of cells with OS vs. PS (Figure 1E), we performed ChIP assay to measure binding of DNMT1 to the promoter regions of these ALDHs. We observed enhanced association of DNMT1 with ALDH2, ALDH3A1, and ALDH6A1 promoters, respectively, in HUVECs under OS vs. PS (Figure 6D-F). The findings suggest that OS induces promoter methylation for ALDH2, ALDH3A1, and ALDH6A1 through promoting the DNMT1 binding.

### Endothelial-specific deletion of Dnmt1 ameliorated the disturbed flow-accelerated atherosclerosis

**Figure 7:**
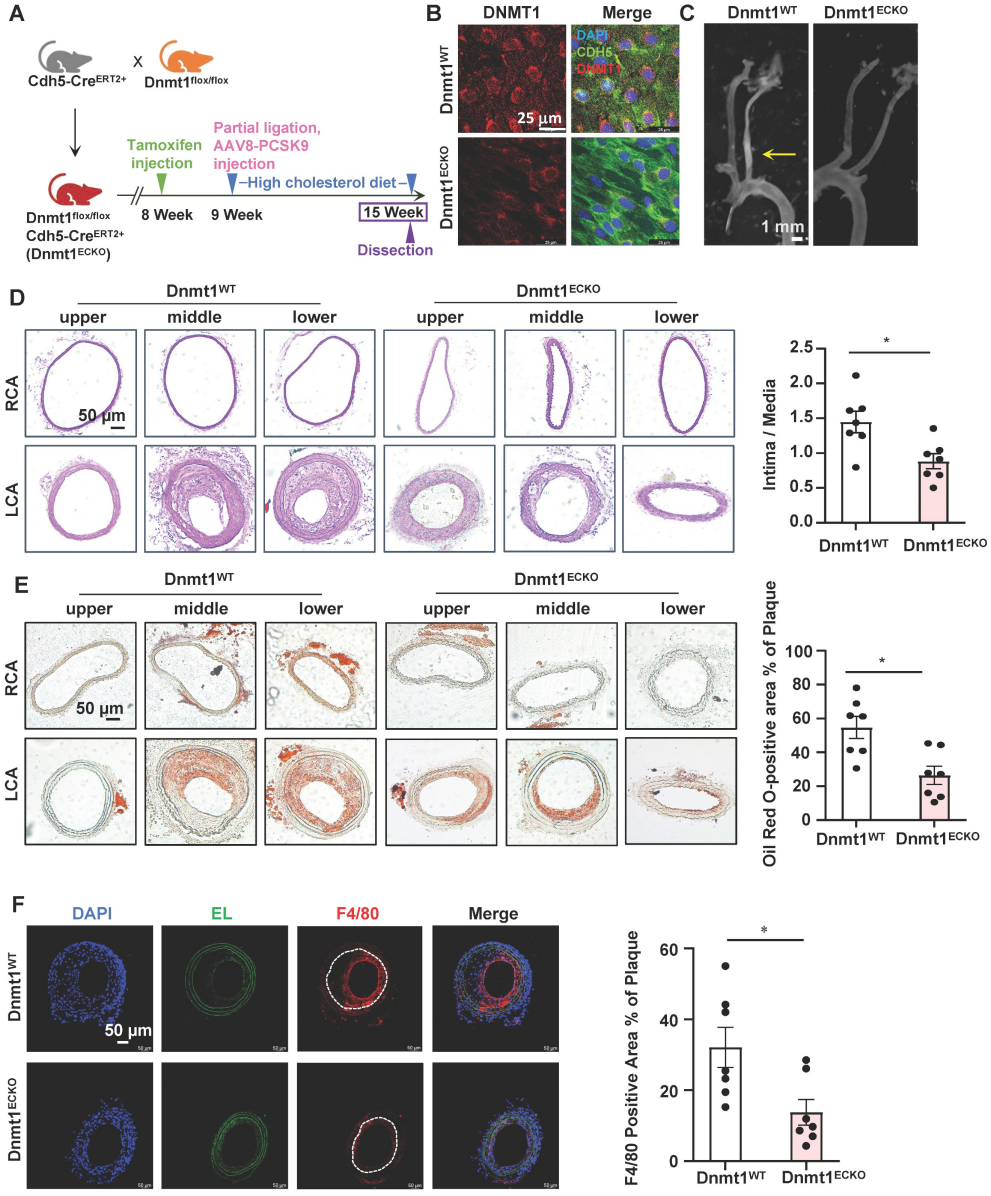
** Endothelial-specific deletion of Dnmt1 ameliorated the disturbed flow-accelerated atherosclerosis. (A)** Schematic diagram of the experimental design in Dnmt1^WT^ and Dnmt1^ECKO^ mice. **(B)** En face immunofluorescence staining with anti-DNMT1 (red) and anti-CDH5 (green) antibodies in the endothelia from thoracic aorta. DAPI (blue) indicates the nuclei. **(C)** Gross images for carotid arteries from the Dnmt1^WT^ or Dnmt1^ECKO^ mice. Plaque are indicated by arrows. **(D-E)** Representative H&E and Oil Red O staining and quantification of plaques in the left common carotid arteries from the Dnmt1^WT^ or Dnmt1^ECKO^ mice. Images were captured from three distinct regions (the upper, middle, and lower regions) of the common carotid arteries. In the graphs, each dot represents the value of the maximum area of the three regions in each mouse. Data were presented as mean ± SEM. *P < 0.05 by Unpaired t test, n = 7. **(F)** Representative immunofluorescent staining of F4/80 (red) indicative of monocyte infiltration in the left common carotid arteries from the Dnmt1^WT^ or Dnmt1^ECKO^ mice. DAPI (blue) indicates the nuclei. White line indicates intima. Data were presented as mean ± SEM. *P < 0.05 by Unpaired t test, n = 7.

EndoMT is known to contribute to the initiation and progression of atherosclerosis 3. To assess the role of endothelial expressing DNMT1 in atherogenesis, we generated inducible EC-specific Dnmt1-deletion (Dnmt1^ECKO^) and the littermate control (Dnmt1^WT^) mice (Figure S9A). Mice were subjected to tamoxifen injection via the intraperitoneal vein (1.5 mg daily per mouse for 7 days) to induce the Cre expression and AAV8-PCSK9 virus once injection via the tail vein followed by a high cholesterol diet to induce hyperlipidemia (Figure 7A). The mice were also subjected to partial ligation of their LCA on the three days before virus administration to introduce disturbed flow into their left common carotid arteries to accelerate atheroma formation in the common carotid arteries (Figure 7A). En face immunofluorescent staining of the arterial inner surfaces from the Dnmt1^ECKO^ and Dnmt1^WT^ mice receiving tamoxifen confirmed that the endothelial DNMT1 expression was almost undetectable in the Dnmt1^ECKO^ mice (Figure 7B). Successful knockout of endothelial Dnmt1 was also confirmed by isolation of mouse endothelial cells for quantitative RT-PCR detection (Figure S10). The body weight, serum lipid level, and blood pressure were comparable between the Dnmt1^ECKO^ and Dnmt1^WT^ mice (Figure S9B-D). At 6-week post-ligation, mice were sacrificed, and aortas/arteries were collected for assessment of atherosclerosis. Gross images for carotid arteries showed that the Dnmt1^ECKO^ mice exhibited a notably reduced formation of atherosclerotic plaques, compared with the Dnmt1^WT^ mice (Figure 7C). H&E and Oil Red O staining of the cross-sections indicated that the LCA from Dnmt1^WT^ mice exhibited marked neointimal thickening and lipid accumulation, which were greatly inhibited in the Dnmt1^ECKO^ mice (Figure 7D-E). Moreover, monocyte infiltration as indicated by the ratio of F4/80+ areas over the neointimal areas was reduced in the Dnmt1^ECKO^ mice, compared with that in the Dnmt1^WT^ mice (Figure 7F). Taken together, these findings suggest that endothelial Dnmt1 positively contributes to the disturbed flow-accelerated atherogenesis.

## Discussion

Endothelial dysfunction plays a critical role in the initiation and progression of atherosclerosis. Dysfunctional ECs can adopt myofibroblast- and SMC-like properties through EndoMT, during which the expression of endothelial markers and functions are lost, and the expression of mesenchymal cell marker and functions acquire 3. Hemodynamic flow disturbance has been demonstrated to induce EndoMT 51-55. The underlying molecular mechanisms are complex and involve a plethora of interconnected pathways. Among the mechanisms and pathways, epigenetic regulation via DNA methylation has been suggested to be a key mechanism involved in shear stress-regulated EndoMT, as evidenced by the findings showing that therapeutic interruption for DNMT expression and activity by using a demethylating agent, 5-Aza, inhibited the disturbed flow-induced EndoMT 13.

Echoing the prior findings, results from our current study also support DNA methylation to be a major player in mediating EndoMT caused by flow disturbance. Notably, we provided important *in vivo* data to consolidate the concept by the use of two animal models including the endothelial lineage tracing mice and the conditional EC Dnmt1 knockout mice, both of which were subjected to partial ligation of carotid to surgically generate disturbed blood flow. Despite that studies have shown the application of lineage tracing system to EndoMT research and proved it a powerful tool 56 and that a very recent work studied the disturbed flow-induced EndoMT in arteriovenous fistula by using the lineage tracing mice 55, our present study further demonstrated an epigenetic mechanism involved in the flow-regulated EndoMT by the utilization of the mice. Moreover, although we and others have previously shown a role of DNMT1 in mediating the disturbed flow-initiated endothelial dysfunction 15, 19-21, no study has been reported related to the utilization of conditional EC Dnmt1 knockout mice in the literature. By using the EC-specific Dnmt1 mutant mice, our study has added a determinant piece of evidence for validation of the role of Dnmt1 in mediating EC dysfunction and atherosclerosis.

Carnosine is a naturally occurring dipeptide that has been considered a useful therapeutic agent playing protective roles against EC damage, due to its anti-glycating and antioxidant activities 57, 58. Carnosine also facilitates nitric oxide production in ECs to produce a vasodilatory action 59. Clinical application of Polaprezinc, a chelated zinc-carnosine drug that has gastric-mucosal-protective activity, improved cardiac function of patients after myocardial infarction 60. However, the detailed mechanisms related to the endothelial and cardiovascular beneficial actions of carnosine and its precursor, β-alanine, are far from being well understood. Here, by analyzing the published datasets from Hanjoong Jo laboratory 15, a potential of β-alanine in the shear-regulated and DNMT-mediated transcriptional changes was proposed. Our results further indicated that combined supplementation of ECs with carnosine and acetyl-CoA in the form of acetate inhibited the shear- or TGF-β1-induced EndoMT. This finding provides a mechanistic explanation for why carnosine, as well as acetyl-CoA, produces an endothelial protective effect. In addition, our data uncovered a molecular basis that supplementation with carnosine and acetate suppressed the activation of Smad2/3, whose activation is proved to be a potent inducer for EndoMT. Although how carnosine and acetyl-CoA prohibit the phosphorylation of Smad2/3 remains to be elucidated, some possibilities driven by prior studies may be considered. For example, carnosine may alter the cellular redox status to inhibit Smad3 activation 61, and acetyl-CoA downregulated the expression of Smad7 to suppress the activation of Smad2/3 25.

The ALDH family proteins ALDH2, ALDH3A1, and ALDH6A1 play pivotal role in β-alanine metabolism in terms of the synthesis and conversion of β-alanine, carnosine, and acetyl-CoA 37. Functions of these proteins in vascular health and disease are insufficiently known. ALDH2 is the best studied subunit of this family and it was suggested to exert a protective role in the endothelium against age-associated dysfunctions to prevent atherosclerosis 62. ALDH3A1 is inadequately studied in cardiovascular system despite of a recent paper showing association of ALDH3A1 with angiogenesis 63. The function of ALDH6A1 has never been reported in vascular endothelium, but a microarray-based experiment showed that the expression of ALDH6A1 was downregulated in atherosclerotic aortas from hyperlipidemia mice compared to the control mice 64, suggestive of the potential involvement of ALDH6A1 dysregulation in atherosclerosis. Our data clearly showed that the endothelial expression of ALDH2, ALDH3A1, and ALDH6A1 was downregulated by atheroprone disturbed flow, both *in vitro* and *in vivo*, implying that these genes might participate in the regulation of endothelial function by shear flow. Importantly, we also identified a regulatory mechanism that disturbed flow causes this downregulation thought DNMT1-mediated DNA methylation on those genes. Furthermore, our loss-of-function study indicated that insufficient expression of any of these genes or all of them caused endothelial dysfunction, i.e., loss of endothelial markers and gain of mesenchymal markers, promotion in cell migration and proliferation, and impairment in endothelial intercellular junctions. It is to be emphasized that the present study is unique in reporting the beneficial role of ALDHs, in particular, ALDH3A1 and ALDH6A1, in regulating endothelial homeostasis.

Despite its contributions, this study has some limitations that warrant consideration. First, to mitigate sex bias, male and female mice were combined for analysis. Nevertheless, existing research from various groups has revealed notable sex differences in ECs under pathological conditions 65, 66. For instance, a study investigating atherosclerosis in EC-lineage tracing ApoE knockout mice found that females exhibited less EndoMT at the advanced stages of atherosclerosis 67. Thus, future investigations exploring the gender disparities in flow-regulation of EndoMT are essential. Second, although the utilization of Tek-Cre mice to generate EC-lineage tracing EndoTrack^EGFP^ mice has been a standard approach 68, recent advancements in cell lineage-tracing studies, particularly when coupled with single-cell RNA sequencing, have prompted a paradigm shift in cell-specific investigations. Consequently, considering the CDH5-Cre mouse model could serve as a viable and more contemporary alternative. Third, this study relied on EC-lineage tracing EndoTrack^EGFP^ mice to explore the impact of disturbed flow on endothelial function through EndoMT. Additionally, Dnmt1 endothelial-specific knockout mice were employed to investigate the role of DNMT1 in atherogenesis. Nonetheless, to enhance the comprehensiveness of the study, the introduction of an EC-specific knockout of Dnmt1 with a lineage tracing component would be beneficial.

In summary, our results demonstrated an importance of ALDH2, ALDH3A1, and ALDH6A1 and the associated β-alanine and carnosine metabolism in preventing atheroprone disturbed-blood-flow-induced EndoMT. Our data suggested a mechanism that disturbed flow or OS decreases the transcription of these ALDHs via promoting binding of DNMT1 to the promoter regions of those genes to cause hypermethylation and gene silencing, triggering the initiation of EndoMT. Our study also reveals that supplementation with carnosine and acetyl-CoA exerted endothelial protective effect, probably through the inhibition on the activation of the TGF-β downstream effector Smad2/3.

## Supplementary Material

Supplementary figures and tables.
