# Supplementary material for: DNMT1 mediates the disturbed flow-induced endothelial to mesenchymal transition through disrupting β-alanine and carnosine homeostasis
Source: Theranostics. 2023 Aug 6;13(13):4392–411. doi: 10.7150/thno.84427 (PMC10465216; doi:10.7150/thno.84427)
Supplement: Supplementary file 1 — Supplementary figures and tables. [file thnov13p4392s1.pdf]

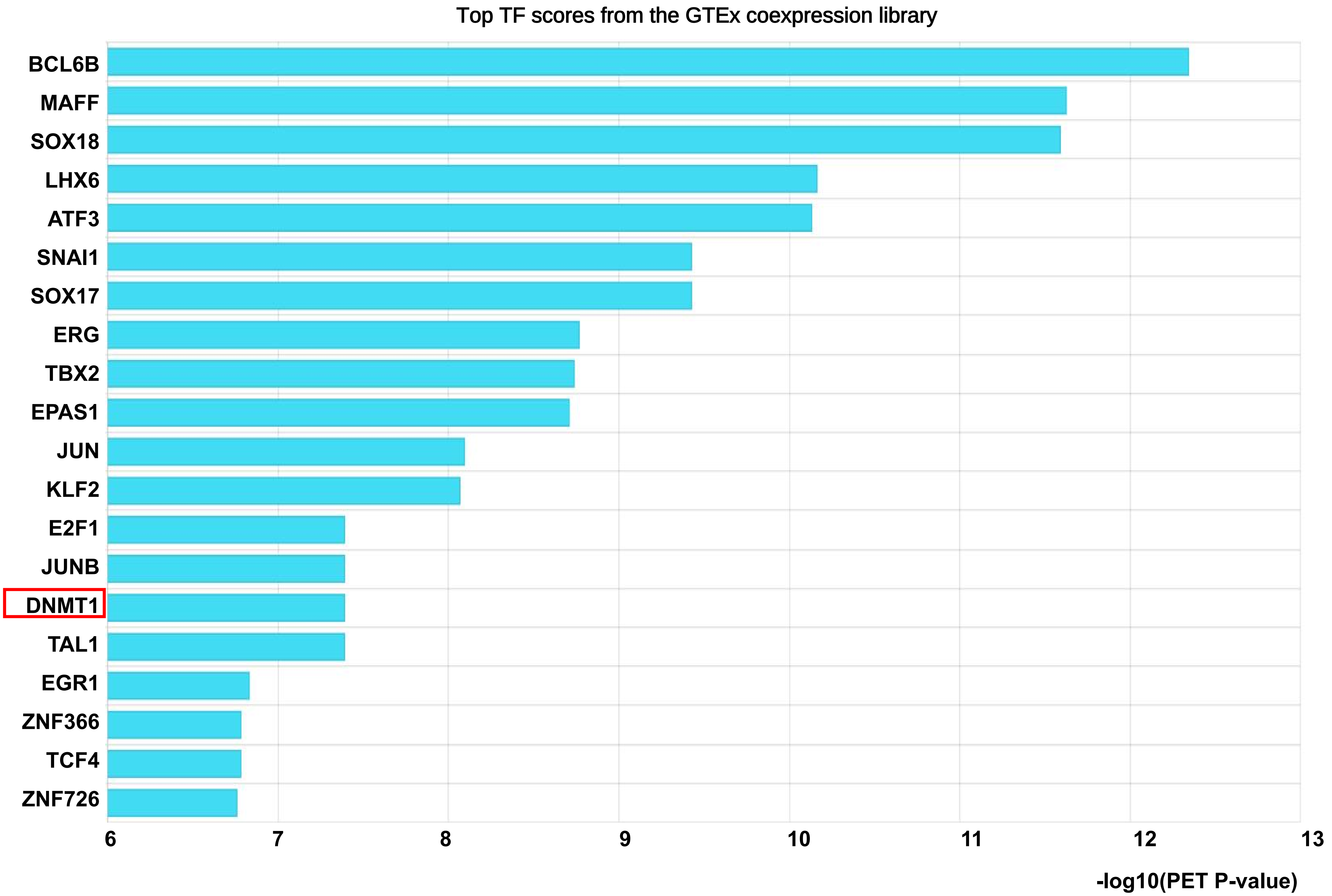

Figure S1. Transcription factor enrichment analysis. The 458 overlapping genes between Dunn et al.'s microarray dataset (genes differentially expressed between the ligated left carotid artery and the unligated right carotid artery) and our laboratory's RNA-seq dataset (genes differentially expressed between OS and PS) were obtained. The 458 overlapping genes were analyzed by using ChEA3 to generate the predicted enrichment of transcription factors.

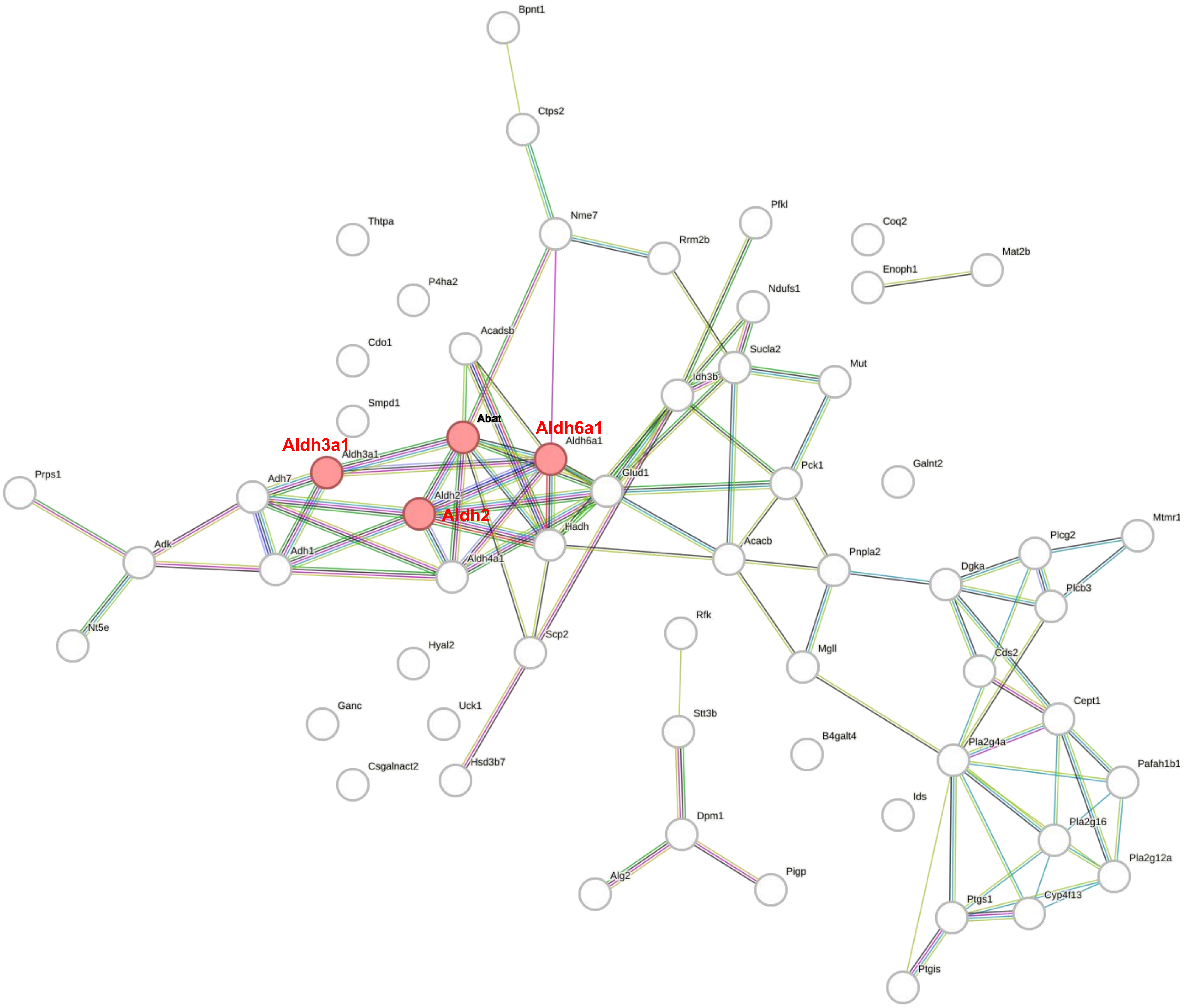

Figure S2. The protein-protein interaction (PPI) network based on STRING database of studies genes. Genes enriched in the metabolic pathways (in Figure 2B) were subjected to analysis of PPI by using the STRING web server. The network nodes indicate genes. The red notes show genes in the β-Alanine metabolic pathway. A green line: neighborhood evidence; a blue line: cooccurrence evidence; a purple line: experimental evidence; a yellow line: textmining evidence; a black line: coexpression evidence.

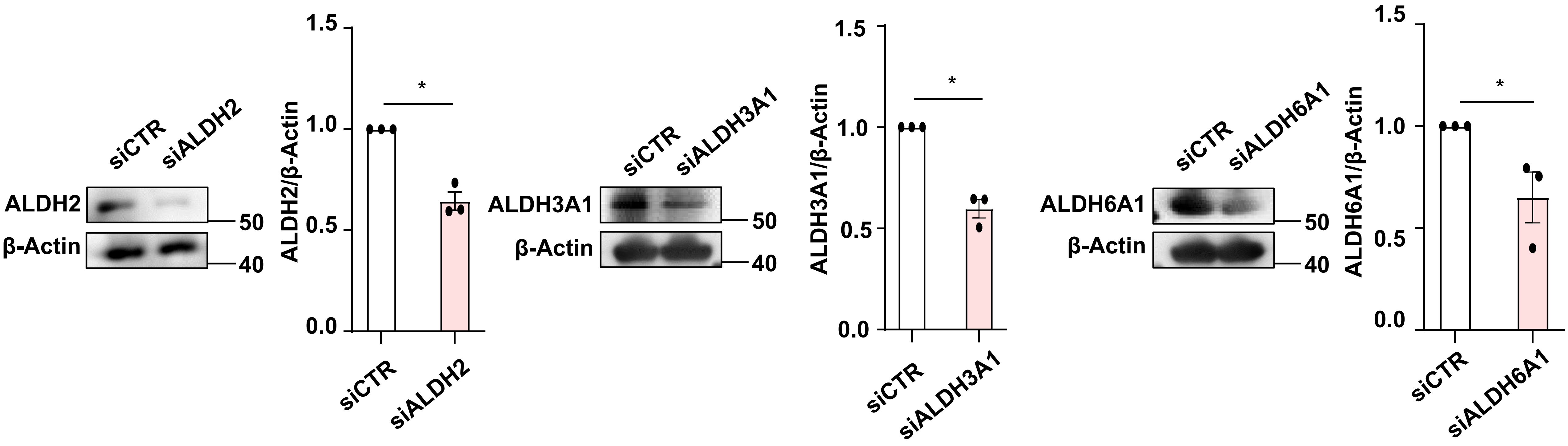

Figure S3. ALDH2, ALDH3A1 and ALDH6A1 knockdown efficiency detection. HUVECs were transfected with ALDH2-specific siRNA (siALDH2) or ALDH3A1-specific siRNA (siALDH3A1) or ALDH6A1-specific siRNA (siALDH6A1) or the control siRNA (siCTR). Western blotting assay was performed to measure the expressions ALDH2, ALDH3A1 and ALDH6A1. Data were presented as mean ± SEM. *P < 0.05 by Unpaired t test, n = 3.

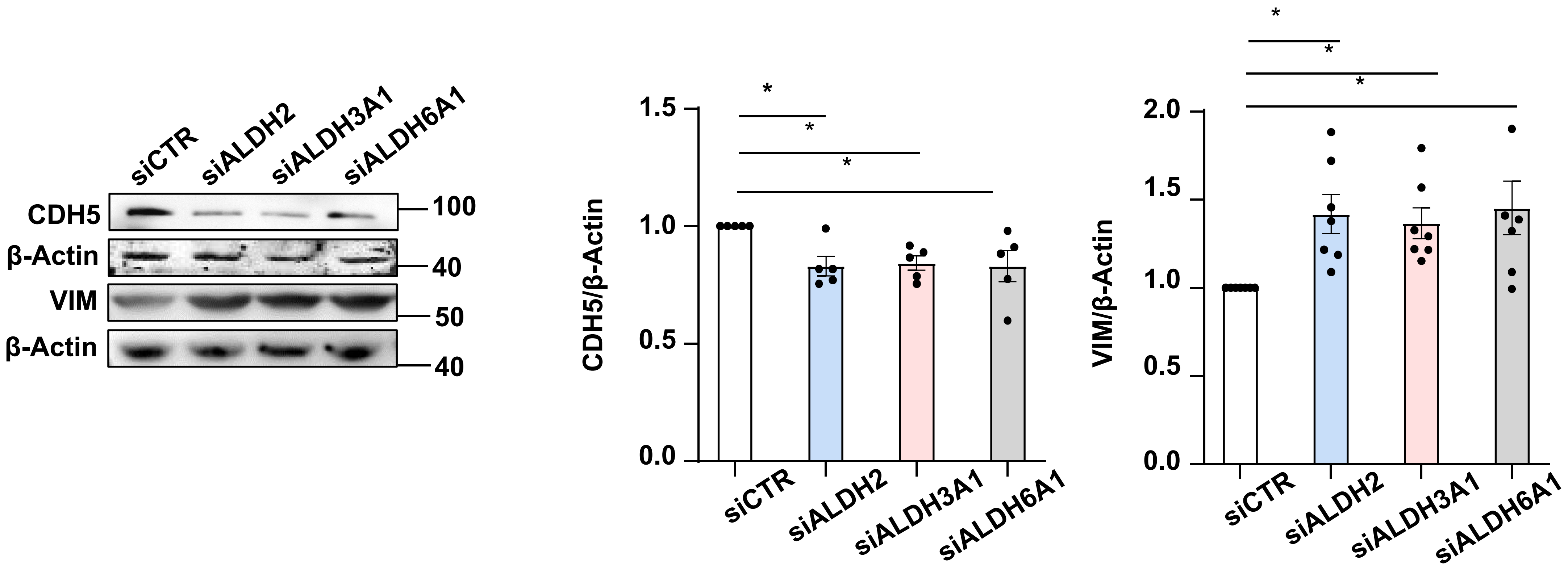

Figure S4. Knocking down ALDH2, ALDH3A1 and ALDH6A1 in HUVECs individually induce EndoMT. HUVECs were transfected with ALDH2-specific siRNA (siALDH2) or ALDH3A1-specific siRNA (siALDH3A1) or ALDH6A1-specific siRNA (siALDH6A1) or the control siRNA (siCTR). Western blotting assay was performed to measure the expressions of CDH5 and VIM. Data were presented as mean ± SEM. **P* < 0.05 by one-way ANOVA followed by Dunnett's multiple comparisons test, n = 5 or 7.

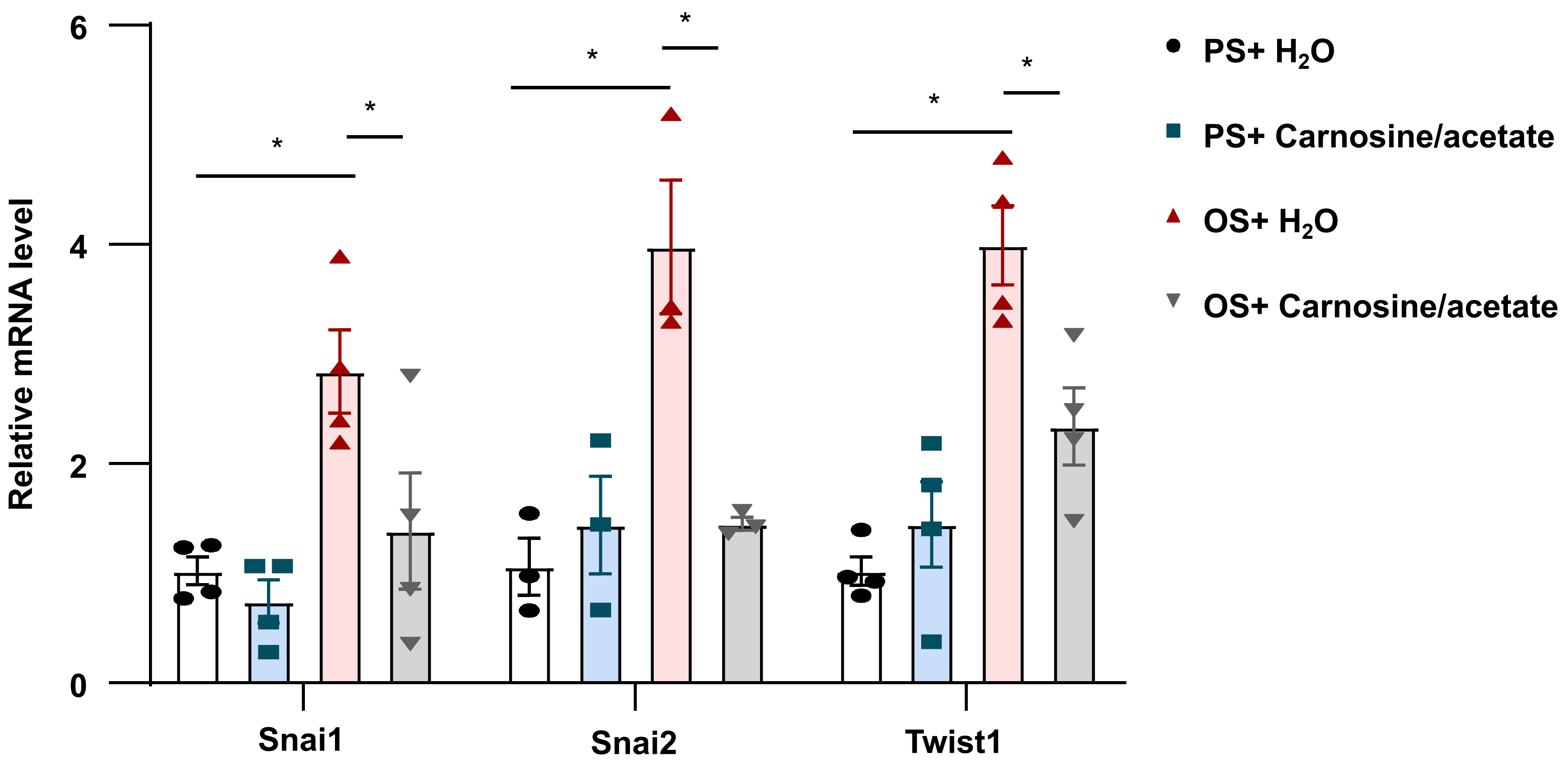

Figure S5. Carnosine and Acetyl-CoA inhibit the increased expression of Snai1, Snai2 and Twist1 caused by OS. HUVECs were pretreated with sterile water or carnosine (500 μmol/L) and acetate (4 mmol/L) for 24 hours, exposed to PS or OS for 24 hours, and quantitative RT-PCR was used to measure the expressions of Snai1, Snai2 and Twist1. Data were presented as mean ± SEM. *$P < 0.05$ by two-way ANOVA followed by Tukey's multiple comparisons test, n = 3, 4.

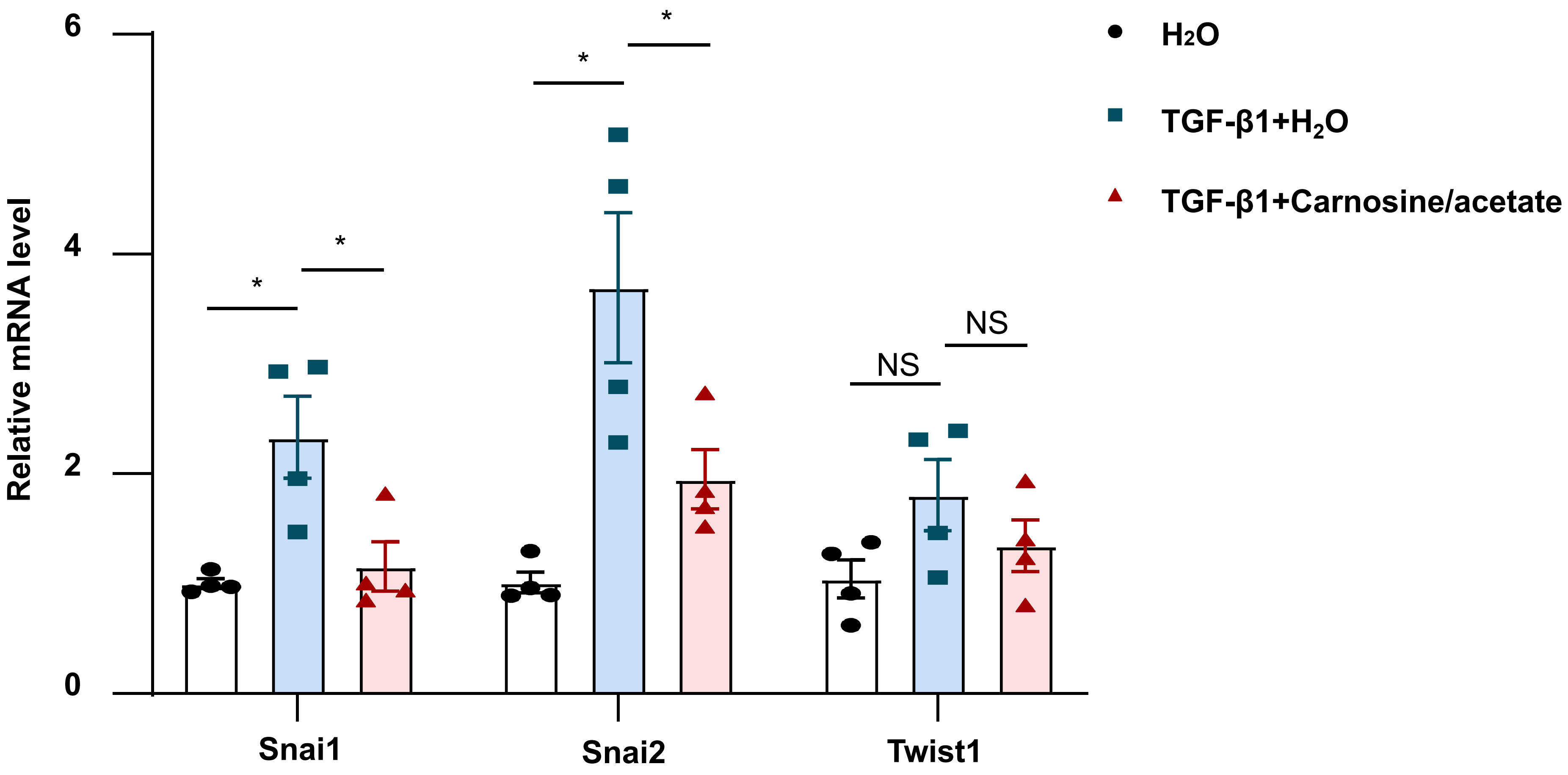

Figure S6. Carnosine and Acetyl-CoA inhibit the increased expression of Snai1 and Snai2 caused by TGF-β1. HUVECs were treated with TGF- β1 (10 ng/mL) for 4 days, and then carnosine (500 μmol/L) and acetate (4 mmol/L) or sterile water were added to the culture medium on the third day; 24 hours later, quantitative RT-PCR was used to measure the expressions of Snai1, Snai2 and Twist1. Data were presented as mean ± SEM. *$P < 0.05$ by one-way ANOVA followed by Tukey's multiple comparisons test, n = 4.

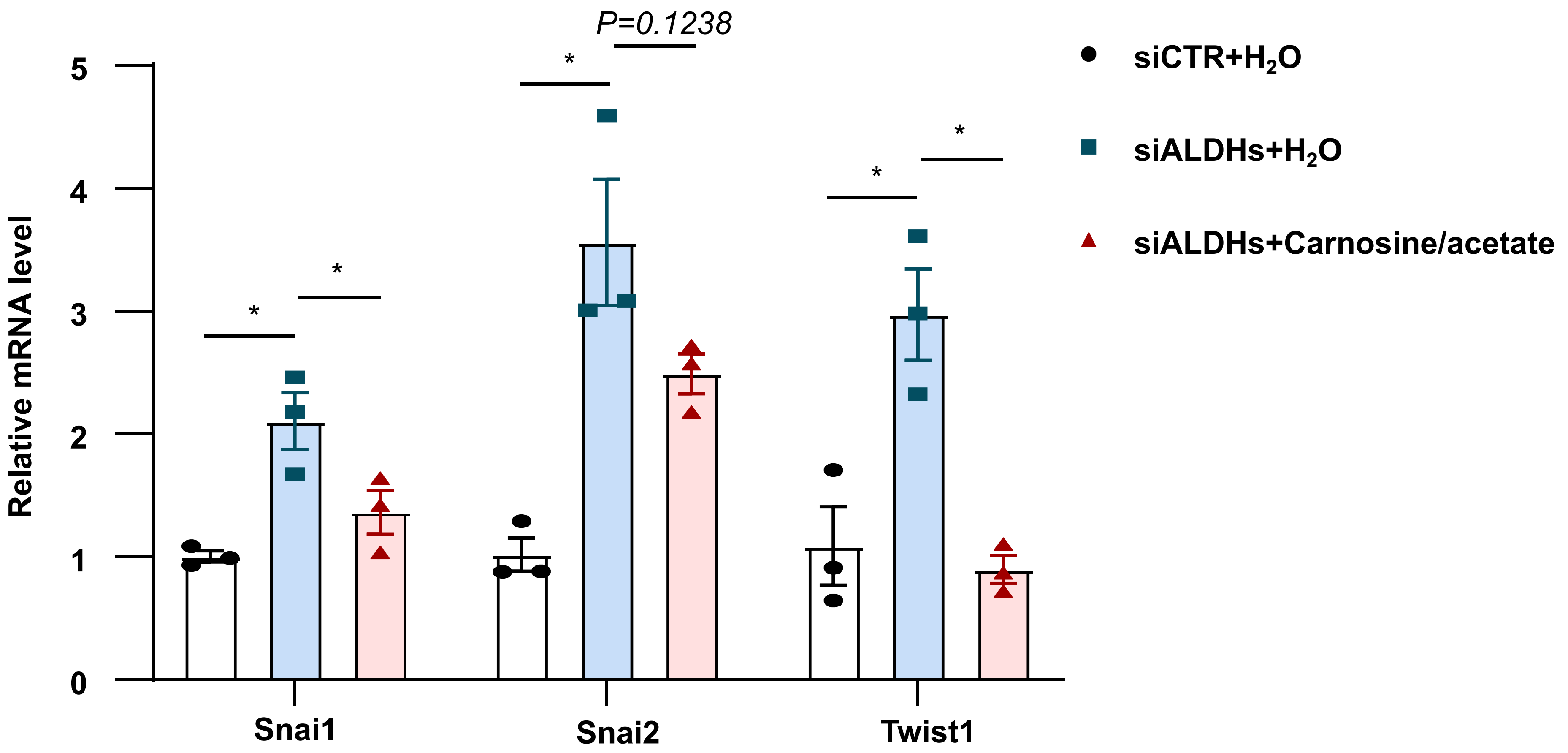

Figure S7. Carnosine and Acetyl-CoA inhibit the increased expression of Snai1 and Twist1 caused by ALDH2/3A1/6A1 knockdown. HUVECs were transfected with siALDH2, siALDH3A1 and siALDH6A1 or siCTR; 48 hours later, carnosine (500 μmol/L) and acetate (4 mmol/L) or sterile water were added to the culture medium. 24 hours later, quantitative RT-PCR was used to measure the expressions of Snai1 , Snai2 and Twist1. Data were presented as mean ± SEM. **P* < 0.05 by one-way ANOVA followed by Tukey's multiple comparisons test, n =3.

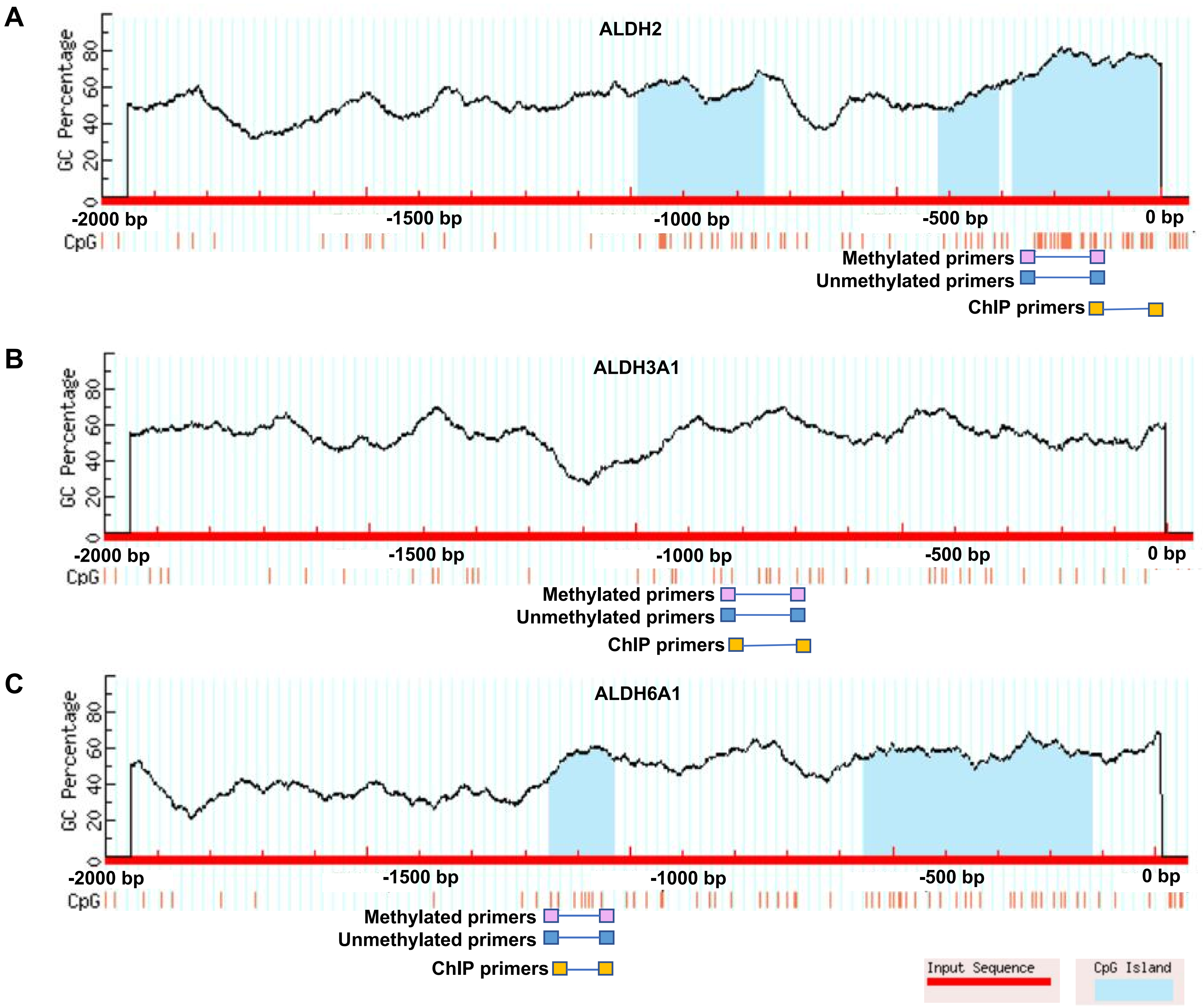

Figure S8. Schematic representation of GpC percentage of the putative promoter regions of human ALDH2, ALDH3A1, and ALDH6A1. Locations of the primer sets for the chromatin immunoprecipitation (ChIP) assay are indicated. The promoter sequences of ALDH2 **(A)**, ALDH3A1 **(B)**, and ALDH6A1 **(C)** were obtained from the UCSC website. CpG island prediction and design for methylated and unmethylated primers were conducted by using the Methprimer web server.

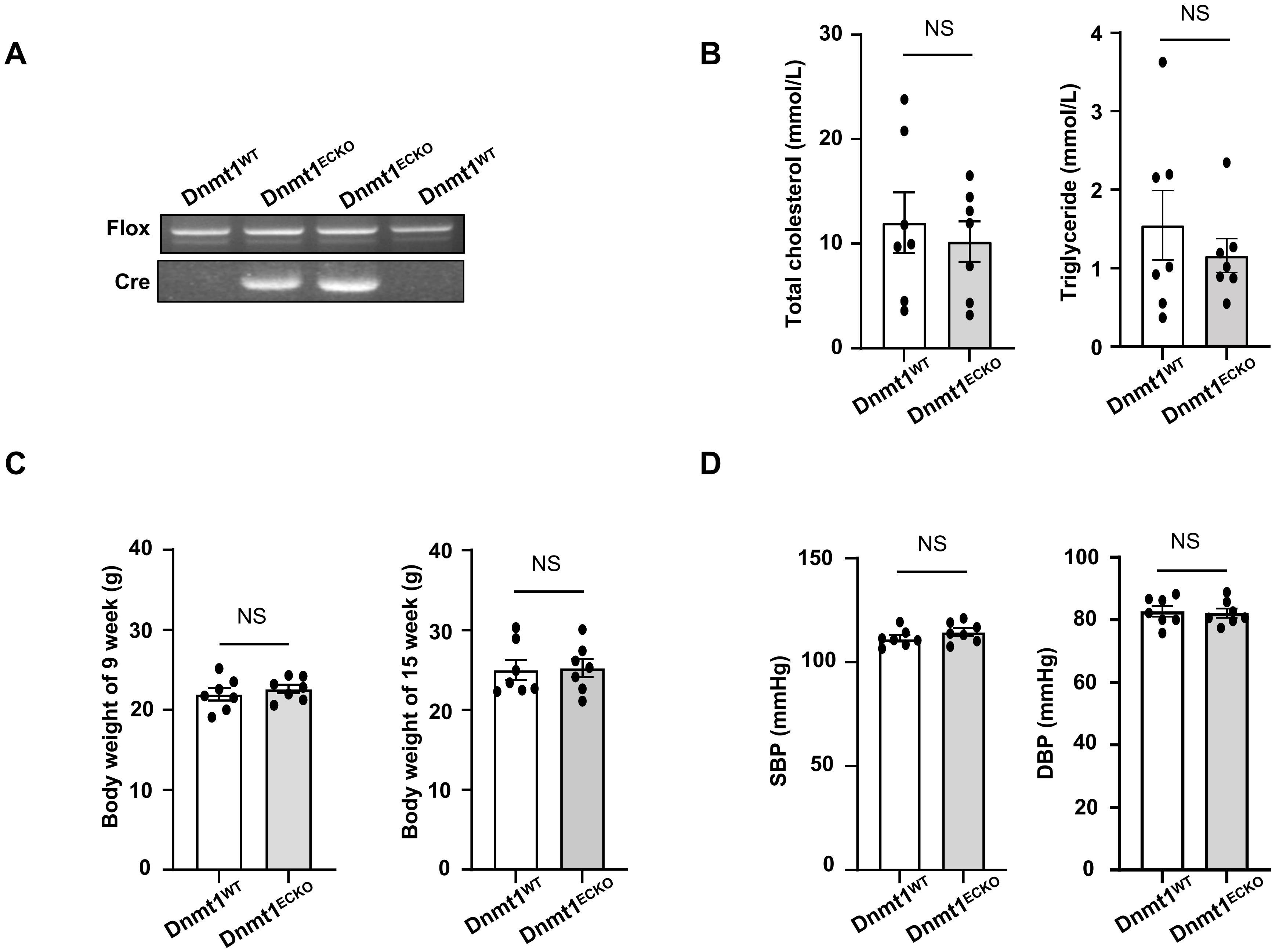

Figure S9. Body weight, blood pressure, and serum lipids in Dnmt1^{WT} and Dnmt1^{ECKO} mice. (A) Representative gel images of PCR products demonstrative of successful generation of Dnmt1^{ECKO} and Dnmt1^{WT} mice. **(B)** Total serum cholesterol, triglyceride level in the Dnmt1^{ECKO} and Dnmt1^{WT} mice at week-15 (n = 7). **(C)** Body weight at week-9 and week-15 (n = 7). **(D)** Systolic and diastolic blood pressures at week-9 (n = 7). SBP: systolic blood pressure; DBP: diastolic blood pressure. Data were presented as mean ± SEM, n = 7. NS: No significance by Unpaired t test.

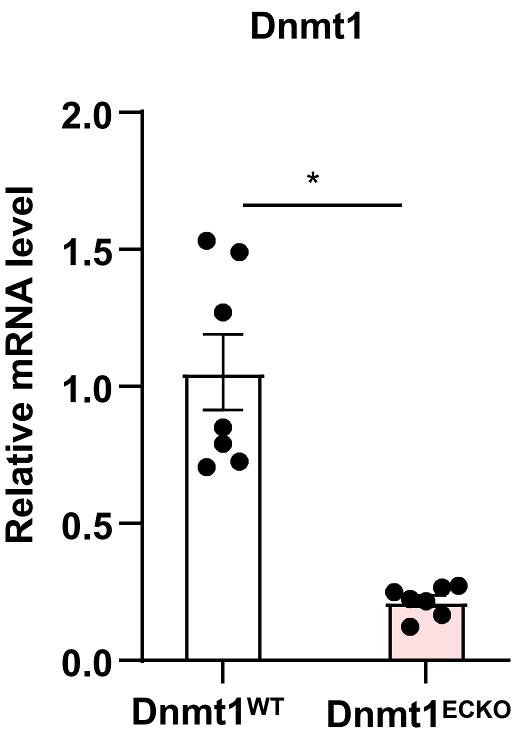

Figure S10. mRNA level detection of Dnmt1 knockout efficiency. Mouse endothelium was collected and Dnmt1 expression was detected by quantitative RT-PCR. Data were presented as mean ± SEM. **P* < 0.05 by Unpaired t test, n = 7.

Table S1. Sequences of the primer

NO.	Primer names	Sequences
1	human siALDH2	5'-AGCCCUAUGUCAUCUCCUAtt-3'
2	human siALDH3A1	5'-CCUGCUACGUGGACAAGAAtt-3'
3	human siALDH6A1	5'-GAGCGAGUCUGUAAUCUGAtt-3'
4	human ChIP-ALDH3A1, forward primer	5'- GGAAGCGCCTGGTGAGAGGG-3'
5	human ChIP-ALDH3A1, rorward primer	5'- GAATAGCTGAGAAGAGGGCC-3'
6	human ChIP-ALDH2, forward primer	5'-TACCTAGCGCCACCCGCTTC-3'
7	human ChIP-ALDH2, rorward primer	5'-AGGGCCACTGAGCCGACCCC-3'
8	human ChIP-ALDH6A1, forward primer	5'-TGTGCAAGACGACACTTAAA-3'
9	human ChIP-ALDH6A1, rorward primer	5'-CCTGTCTCCTCAGTTTGGGA-3'
10	human ALDH6A1, forward primer	5'-GGCAGACACTTCAGTATTAAGCC-3'
11	human ALDH6A1, reverse primer	5'-AGAGGCAGACGGTAGGAATAAA-3'
12	human ALDH2, forward primer	5'-CCTCACCGCCCTCTATGTG-3'
13	human ALDH2, reverse primer	5'-CGGCCAATCTCAGTGGAGC-3'
14	human ALDH3A1, forward primer	5'-TGTTCTCCAGCAACGACAAGG-3'
15	human ALDH3A1, reverse primer	5'-AGGGCAGAGAGTGCAAGGT-3'
16	human TAGLN, forward primer	5'-AAGAATGATGGGCACTACCG-3'
17	human TAGLN, reverse primer	5'-ATGACATGCTTTCCCTCCTG-3'
18	human ACTA2, forward primer	5'-CAGGGCTGTTTTCCCATCCAT-3'
19	human ACTA2, reverse primer	5'-GCCATGTTCTATCGGGTACTTC-3'
20	human VWF, forward primer	5'-CCGATGCAGCCTTTTCGGA-3'
21	human VWF, reverse primer	5'-TCCCCAAGATACACGGAGAGG-3'
22	human CD31, forward primer	5'-GAGTCCAGCCGCATATCC-3'
23	human CD31, reverse primer	5'-TGACACAATCGTATCTTCCTTC-3'
24	human β-Actin, forward primer	5’-CATGTACGTTGCTATCCAGGC-3
25	human β-Actin, reverse primer	5’-CTCCTTAATGTCACGCACGAT-3
26	human CDH5, forward primer	5'-CACCTTCTGCGAGGATATGG-3'
27	human CDH5, reverse primer	5'-AGGAAGATGAGCAGGGTGAT-3'
28	human VIM, forward primer	5'-GCCCTAGACGAACTGGGTC-3'
29	human VIM, reverse primer	5'-GGCTGCAACTGCCTAATGAG-3'
30	human MSP-ALDH6A1-LM	5'-AGATAGGTTGCGATTTGTGTAAGAC-3'
31	human MSP-ALDH6A1-RM	5'-CCTCAATTTAAAACCATTACCGTA-3'
32	human MSP-ALDH6A1-RU	5'-ATAGGTTGTGATTTGTGTAAGATGA-3'
33	human MSP-ALDH6A1-LU	5'-CCTCAATTTAAAACCATTACCATA-3'
34	human MSP-ALDH2A1-LM	5’-TATATATTGGGGGTTTAATTAAGGC-3’
35	human MSP-ALDH2A1-RM	5’-ACTAATACAAACGAAACGAATAACG-3’
36	human MSP-ALDH2A1-RU	5’-TATATTGGGGGTTTAATTAAGGTGA-3’
37	human MSP-ALDH2A1-LU	5’-AATACAAACAAAACAAATAACACT-3’
38	human MSP-ALDH3A1-LM	5’- GATCGGAATTTTGTGTTTTTTTC-3’
39	human MSP-ALDH3A1-RM	5’- CTAAACTCCTACTCTACACCACGCT-3’
40	human MSP-ALDH3A1-RU	5’- GATTGGAATTTTGTGTTTTTTTTG-3’
41	human MSP-ALDH3A1-LU	5’- CTAAACTCCTACTCTACACCACACT-3’
43	human Snai1, forward primer	5’-TCGGAAGCCTAACTACAGCGA-3’
44	human Snai1, reverse primer	5’-AGATGAGCATTGGCAGCGAG-3’
45	human Snai2, forward primer	5’-TGTGACAAGGAATATGTGAGCC-3’
46	human Snai2, reverse primer	5’-TGAGCCCTCAGATTTGACCTG-3’
47	human Twist1, forward primer	5’-GTCCGCAGTCTTACGAGGAG-3’
48	human Twist1, reverse primer	5’-GCTTGAGGGTCTGAATCTTGCT-3’

Table S2. Analysis of microarray data (GEO GSE56143) to show the fold changes of ALDH2, ALDH3A1, and ALDH6A1 gene expression.

	DMSO LCA/RCA	5-Aza LCA/LCA
AIDH2	0.62	1.46
AIDH3A1	0.20	2.57
AIDH6A1	0.48	2.19

The fold change is calculated by the ratio between groups. LCA: left carotid artery; RCA: right carotid artery; 5-Aza: 5-aza-2'-deoxycytidine.
